# Levels of perceived stress according to professional standings among dental surgeons of Karachi: a descriptive study

**DOI:** 10.1186/s12903-022-02272-5

**Published:** 2022-06-18

**Authors:** Maria Khadija Siddiqui, Muhammad Taqi, Shagufta Naqvi, Syed Ali Raza, Hajra Bawany, Zainab Hasan

**Affiliations:** 1 Department of Community Dentistry, Jinnah Medical and Dental College, 22-23, Main Shaheed-e-Millat Road, Karachi, 74800 Pakistan; 2 Department of Community, Preventive Dentistry Dow Dental College, Dow University of Health Sciences, Karachi, Pakistan; 3 Department of Community Health Science, Jinnah Medical and Dental College, Karachi, Pakistan; 4 Department of Community Dentistry, Sir Syed College of Medical Sciences for Girls, Karachi, Pakistan; 5 Department of Dental Material, Jinnah Medical and Dental College, Karachi, Pakistan; 6 Department of Community Medicine, Jinnah Medical and Dental College, Karachi, Pakistan

**Keywords:** Dentists, Pakistan, Professional stress, Stress, Work-related

## Abstract

**Background:**

Globally, occupational stress is a common finding among dentists. The present study aimed to assess prevalence of perceived stress among practicing dentists of Karachi, Pakistan, and assess the perceived stress levels according to the professional standing among dental surgeons.

**Methods:**

A cross-sectional survey was conducted using a convenience sampling technique in which 200 dentists from Karachi were recruited. A self-constructed questionnaire was used to collect data, including demographic and professional backgrounds. Stress level assessment was performed using the perceived stress scale (PSS).

**Results:**

The response rate was 78.5%. In general, a moderate stress level (mean PSS = 18.35 ± 5.417) appeared in the sample size of 157 dentists, and the prevalence of perceived stress was 86%. The level of perceived stress was significantly lower in groups including 40 years old and above (mean diff; *p* = − 0.197), Rupees 1 lac (100,000) and more of monthly income (mean diff; *p* = 0.029), 11 and more years of experience (mean diff; *p* = 0.001) and Assistant Professor/Associate Professor/Professor (mean diff; *p* = 0.035).

**Conclusion:**

All groups representing the senior status of dentists have appeared with lower stress than groups representing the junior status of dentists. Exploratory studies are required to discover an effective coping strategy to deal with occupational stress among the junior dentists of Karachi.

**Supplementary Information:**

The online version contains supplementary material available at 10.1186/s12903-022-02272-5.

## Background

Various occupations have demonstrated an adverse effect on physical and mental health. Psychological stress has appeared as one of the consistent outcomes of occupational hazards in Karachi [1]. Professionals with an occupation in which direct contact with the community is required have demonstrated excessive stress issues. Formerly, dentists remained highest in numbers among professionals who consider their occupation stressful [2]. Common stressors associated with dentistry have previously been reported, including financial issues, the threat of complaints, dissatisfied patients, running behind schedule, and managing complex patients [3, 4]. Dentists in Pakistan exhibit severe stress associated with elective dental procedures and pandemic situations [5]. The current pandemic has further damaged dentists' physical and mental health and their financial situation in recent years. This could be explained as lockdown and fear of getting infected has restricted many dentists in Pakistan from continuing their practices [4, 5]. Stress could manifest as fatigue, sleeplessness, dizziness, gastrointestinal problems, irritability, tachycardia, and cynicism [6]. The sequelae of stress include depression, anxiety, substance misuse, absenteeism, decreased work efficiency, and exhaustion [7]. The professional characteristics of dentists have shown an association with the degree of stress, including experience and the number of qualifications. For instance, in Hong Kong, a higher level of burnout appeared among dentists without post-graduation than dentists with post-graduation [8].

Authors discovered very few studies related to stress done on Pakistani dentists. However, a significant number of dentists appeared to have severe stress in all of those studies [9, 10]. These findings indicate that stress is a health problem among Pakistani dentists. Most studies identified from Karachi were done on dental undergraduates or post-graduate students. According to these studies, female dental students in Karachi are more anxious and depressed than male dental students [11, 12]. Social attitudes and cultural norms that tend to marginalize women in Pakistan place a substantial psychological impact on them which perhaps explains why more females suffer from stress, anxiety and depression than the males in the country [13]. Thus, it is necessary to evaluate further association of gender and other demographic factors with stress, but this time among established dentists in Karachi. Besides that, it is significantly required to find an association between the professional characteristics of dentists and their stress levels. Therefore, the present study aimed to assess the prevalence of perceived stress and identify the association of demographic and professional characteristics with stress levels among practicing dentists.

## Methods

### Research design

A cross-sectional survey was conducted using a convenience sampling technique. Data was collected from two private and two government dental teaching institutions in Karachi from December 2017 to February 2019, along with clinical practices that were convenient to access for data collection. All participants were requested to return the questionnaire immediately after filling it.

### Sample

The sample size for this study was estimated using prevalence-based formula. Using the 86% prevalence of Kay and Lowe’s (2008) study [14] and a confidence limit of 5%, the sample size required for the current study was 185. As a final, 200 dentists from Karachi were recruited to limit the chance of sampling error. Questionnaires were distributed by hand to all participants, irrespective of their gender and age. Dental students and dental graduates who were not working at the time of data collection were excluded from the study as the main idea was to evaluate stress amongst practicing dentists only.

### Instrument

Self-constructed questionnaires were used for data collection. A questionnaire was designed to collect demographic and professional variables related to the standing of the dentists, including age, sex, marital status, working sector, monthly income in Pak rupees (PKR), dental specialty, designation, year of graduation and years of experience.

The designation was ordered according to an ascending academic and clinical rank use in Pakistan for dental professionals. Thus, the designation was labeled as follows: Junior-most level (fresh graduates, working as House Officers), junior level (graduates working as Lecturers or as GDPs [General Dental Practitioner]), midlevel (postgraduate-dental trainees), and lastly, senior-level (all dentists with post-graduation, working as senior faculty members, including, Assistant Professors, Associate Professors, and Professors).

The perceived stress level assessment was performed using Cohen's perceived stress scale-10 (PSS-10) [15]. This tool was originally developed in 1983 and remained a popular choice to understand how different situations affect our feelings and perceived stress [16]. Good reliability and validity of this scale have been reported several times in previous studies, making it acceptable to use in both research and practice (Cronbach's alpha > 0.7) [17]. This scale measures the frequency of experience of the feeling and thoughts during the last month on a given scenario answered as (never, almost never, sometimes, often, very often). By reversing responses (e.g., 0 = 4, 1 = 3, 2 = 2, 3 = 1 and 4 = 0) to the four positively stated items (items 4, 5, 7, and 8) and then summing across all scale items, the PSS scores are derived. PSS scores of 13 are considered average [18]. The level of stress was graded as low scores (0–12), moderate (13–19), and high (≥ 20).

### Statistical analysis

Statistical Package for Social Sciences for Windows was used to analyze the data (SPSS version 21). Frequencies and mean readings were derived using descriptive statistics. The prevalence of stress was calculated by adding moderate and high PSS scores. To check significant mean PSS scores, a t-test and ANOVA were used. Tukey HSD was applied to check significant differences in PSS mean scores between the groups. A *p*-value < 0.05 was considered significant.

The linear regression analysis was used to determine the variables associated with stress in which unadjusted and adjusted *β* coefficients with their 95% *CI*s were reported. Multiple linear regression was used to adjust the results for confounding factors, and a model was created. Three dummy variables 1: House officers, 2: Postgraduate-dental trainees, 3: Lecturers/GDP were formed for the designation, using Assistant professors (AP) and above as reference variable. Gender was introduced along with age, years of experience, working sector, and designations (3 dummy variables) in our final model.

## Results

**Fig. 1:**
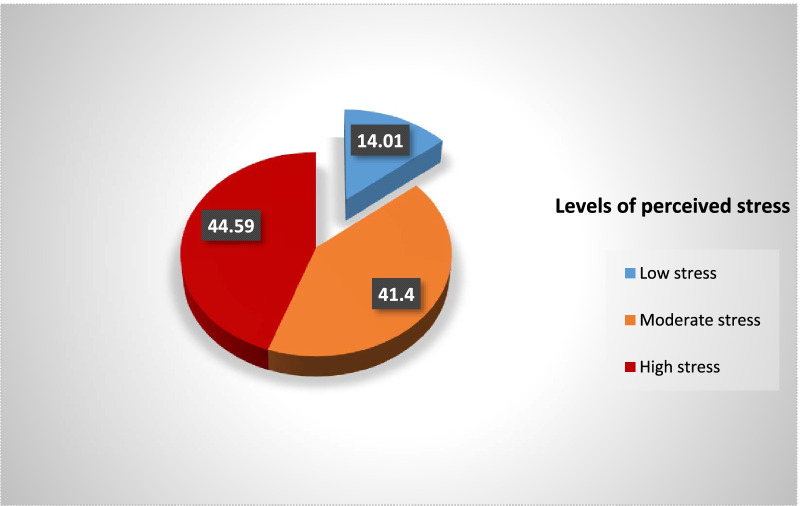
Prevalence of perceived stress in the entire sample of Pakistani dentists (n = 157)

Out of 200 questionnaires, 157 dentists returned the filled questionnaire immediately to the researcher, establishing a response rate of 78.5%. Unfilled and incomplete questionnaires were removed from the study. Frequency of demographic variables and PSS scores are given in Table 1. Majority [73.2% (n = 115)] of respondents were young dentists from Karachi who were 20–29 years old. Mean age of the sample was 28.1 ± 7.51 years and mean PSS score derived was 18.35 ± 5.417. Figure 1 shows the prevalence of perceived stress in all participants, which was 86% (n = 135), comprising high-level 44.6% (n = 70) and moderate level 41.4% (n = 65) of stress. According to the results, only 14% (n = 22) of dentists have perceived low-level stress. The comparison of mean PSS scores for all demographic and professional variables are shown in Table 2. All variables have shown significant mean PSS scores, except for gender and specialty. The highest mean PSS score of 21.67 ± 6.910 (*p* = 0.014) was observed for dentists working without pay (honoree group), followed by government employees, 20.39 ± 4.692 (*p* = 0.010). An additional file shows frequency of all variables and PSS scores in more detail [see Additional file 1].

**Table 1:** Frequencies of demographic variables and levels of stress among a sample of Pakistani dentists (n = 157)

Variable	(Mean ± SD)	Median	N (157)	%
Age in years	28.1 ± 7.51	26.0(6)		
20–29			115	73.2
30–39			29	18.5
40–49			7	4.5
50 and above			6	3.8
Gender	–	–		
Male			55	35
Female			102	65
Marital status	–	–		
Married			57	36.3
Single			100	63.7
Working sector	–	–		
Private			121	77.1
Government			36	22.9
Stress	18.3 ± 5.41	18.0(6)		
Low			22	14.01
Moderate			65	41.4
High			70	44.59

**Table 2:** Comparison of mean PSS scores between all demographic and professional characteristics among a sample of Pakistani dentists (n = 157)

Variable	Mean ± SD	*p*-value	Total n = 157
*Gender*			
Female	18.85 ± 5.842	0.086	102
Male	17.42 ± 4.425		55
*Marital status*			
Married	16.72 ± 5.421	0.004*	57
Single	19.28 ± 5.217		100
*Work sector*			
Government	20.39 ± 4.692	0.010*	36
Private	17.74 ± 5.487		121
*Specialty*			
Non-specialist	18.79 ± 4.646	0.262	90
Specialist	17.76 ± 6.296		67
*Age*			
20–29	19.01(5.428)	0.008*	115
30–39	17.55(4.702)		29
40 and above	14.31(5.154)		13
*Years of experience*			
Up to 5 years	19.29 ± 5.230	0.002*	92
6 to 10 years	18.72 ± 4.250		29
≥ 11 years	15.64 ± 5.934		36
*Designation*			
House officers	18.93 ± 5.099	0.015*	60
Lecturer/GDPs	18.75 ± 5.336		57
Postgraduate-dental trainees	18.67 ± 4.724		24
~ Asst./Asso./Prof	14.25 ± 6.506		16
*Monthly income in PKR*			
Honoree (no pay)	21.67 ± 6.910	0.014*	09
5000–19,000	19.29 ± 4.539		42
20,000–59,000	18.12 ± 5.003		66
60,000–99,000	18.73 ± 4.891		22
100,000 and above	14.89 ± 7.128		18

*p-value < 0.05 was considered significant

~ Asst. = Assistant Professor, Asso. = Associate Professor, Prof. = Professor

Significant differences in mean PSS scores between groups within significant categories are shown in Table 3. In the case of age, 20–29 years old dentists demonstrated significantly higher stress than those 40 years old and above (*p* = -0.197). In terms of experience, dentists with up to 5 years of experience showed significantly higher PSS scores than dentists with ≥ 11 years of experience (*p* = 0.001). The difference in mean PSS scores was significant between dentists working as honorees (without pay) (*p* = 0.017) and dentists who monthly earn 1 lac (100,000) PKR and more. Similarly, difference in mean PSS scores was significant between dentists with a low monthly salary of 5000–19,000 PKR (*p* = 0.029) and dentists who monthly earn 1 lac (100,000) PKR and more. In terms of designation, house officers reported a significantly higher mean PSS score than the Professors (*p* = 0.035).

**Table 3:** The significant difference in mean PSS scores within categorical groups; (age and professional characteristics) among a sample of Pakistani dentists (n = 157)

Groups with the significant difference in stress level	N	PSS score Mean ± SD	F statistics (df)	*p*-value
*Age in years*				
20–29	115	19.01 ± 5.428	F = 5.032 (2154)	− 0.197
40 and above	13	14.31 ± 5.154		
*Years of experience*				
Up to 5 years	92	19.29 ± 5.230	F = 6.386 (2154)	0.001
≥ 11 years	36	15.64 ± 5.934		
*Monthly income in (PKR)*				
5000–19,000	42	19.29 ± 4.539	F = 3.224 (4152)	0.029
100,000 and above	18	14.89 ± 7.128		
Monthly income in thousands (PKR)				
Honoree (no pay)	09	21.67 ± 6.910	F = 3.224 (4152)	0.017
100,000 and above	18	14.89 ± 7.128		
*Designation*				
House officers	60	18.93 ± 5.099	F = 3.591 (3153)	0.035
~ Asst./Asso./Prof	16	14.25 ± 6.506		

Categories with only significant differences are shown above

A *p*-value of < 0.05 was considered significant

~ Asst. = Assistant Professor, Asso. = Associate Professor, Prof. = Professor

The simple linear regression showed significant association of PSS score with independent variables of age (continuous variable; *p* = 0.013*), marital status (*p* = 0.004*), working sector (*p* = 0.010*), designation of lecturer/GDP (*p* = 0.010*) and years of experience (*p* = < 0.001**) as shown in Table 4

**Table 4:** Simple linear regression analysis of the PSS (perceived stress scale) and its associated factors among a sample of Pakistani dentists (n = 157)

Variables	Beta coefficient	*p*-value	95% CI	R^2^
Age	− 0.142	0.013*	− 0.254, − 0.030	0.039
Years of experience	− 0.208	< 0.001**	− 0.310, − 0.107	0.096
Gender	1.435	0.114	− 0.347, 3.216	0.016
*Female*				
Marital status single	2.561	0.004*	0.826, 4.295	0.052
Work sector government	2.645	0.010*	0.651, 4.639	0.42
*Designation 1*				
House officers with AP above	0.944	0.290	− 0.813, 2.700	0.007
*Designation 2*				
Post graduate trainee with AP above	0.373	0.757	− 2.007, 2.754	0.001
*Designation 3*				
Lecturer with AP above	0.634	0.482	− 1.144, 2.413	0.003
Monthly income	− 1.252	0.002*	− 2.046, − 0.458	0.06

*p < 0.05; **p < 0.001

In Multiple Linear regression, the predictors of stress in the study participants were found to be age (β = 0.528, *p* = 0.002*), female gender (β = 0.173, *p* = 0.032*), years of experience (β = − 0.683, *p* = < 0.001*)*, government sector (β = 0.225, *p* = 0.004*) and designation (lecturer vs AP and above) (β = 0.371*, p* = 0.013) as shown in Table 5.

**Table 5:** Multiple linear regression analysis of the PSS (perceived stress scale) and its associated factors among a sample of Pakistani dentists (n = 157)

Variable	Standardized Coefficients Beta (β)	*p*-value	95% CI
Constant	6.150		
Age (years)	0.528	0.002*	0.146, 0.614
Years of experience (years)	− 0.683	< 0.001**	− 0.663, − 0.256
Gender (female gender)	0.176	0.032*	0.178, 3.801
Working sector (Government)	0.225	0.004*	0.934, 4.837
Designation House officers with AP and above	0.194	0.270	− 1.893, 6.015
Designation Post Graduates trainees with AP and above	0.190	0.151	− 1.049, 6.745
Designation Lecturers/GDPs with AP and above	0.371	0.013*	0.907, 7.416

R^2^ = 0.218; adjusted R^2^ = 0.182; df = 7149; F statistics = 5.949; CI = Confidence interval of B (*p < 0.05; **p < 0.001)

## Discussion

The main idea behind the current study was to explore a new aspect of stress levels associated with demographic and professional standing. Interestingly, in the present study, all groups representing a senior status of dentists, including 40 and above age, highest range of income, most years of experience, and highest designation, have appeared with lower stress than groups representing the junior status of dentists. Hence, establishing a shred of evidence that with growing age, income, experience, and higher designation, the level of stress decreases among dentists.

In our study, a significant proportion of dentists demonstrated stress, favoring many previous international studies, thus, highlighting the stressful nature of dentistry worldwide [19–23]. Although stress levels in female dentists were slightly higher (18.85 5.842) than in males (17.424.425), there was no significant difference in mean PSS scores between the two genders. Similarly, Jahi and Pouradeli found no significant differences in stress levels between male and female dentists [21, 22].

Female gender was confirmed as a predictor of stress in a previous study conducted on Pakistani dentists [5], whereas in current study, female gender appeared as an insignificant factor in the linear regression. However, when gender was included in the multiple regression model alongside age, years of experience, working sector, and designations (3 dummy variables), female gender turned out to be significantly associated with stress.

In the current study, age was found to have a significant relationship with stress in both simple and multiple regression. These findings are consistent with the findings of a previous study of Pakistani dentists [5], in which age of dentists had a significant association with stress in both simple and multiple regression. In our study, young dentists (20–29 years old) experienced significantly more stress than senior dentists (40 and above). Our findings are similar to those of Yemeni and Indian studies, where young dentists appeared to be more stressed [24, 25]. These findings suggest that young dentists may face more uncertainty as a result of their lack of practical experience, leading to anxiety and stress.

According to a study, post-graduate trainee doctors from public sector universities in Pakistan appeared to be more stressed than those from private sector universities in Pakistan [26]. Resembling this scenario of Pakistani doctors, in our study, dentists employed in government sectors have shown significantly higher stress levels (mean: 20.39 ± 4.692) than dentists employed in private sectors (mean: 17.74 ± 5.487). Moreover, employment in the government sector has emerged as an independent and confounding factor significantly associated with stress in dentists.

The current study has discovered a decent proportion of dentists working without pay (Honoree), signifying a high demand for dental jobs in Karachi. Furthermore, their mean PSS score was significantly higher than that of the dentists who were paid. The lowest stress was observed for dentists with the highest income in the study. These findings are in line with the studies conducted on Romanian [27] and Korean dentists [23, 28], where high income was found to have a negligible relationship with mental stress.

Years of experience were found to have a significant negative correlation with stress among dentists in the current study. Similarly, few previous studies [29, 30] found a negative relationship between dentists’ years of experience and stress. According to our study, the mean PSS scores have declined with more years of experience and increased with fewer years of experience. These findings are consistent with the findings of a study by Maroof, which identified a similar scenario of Pakistani health care workers based on work experience [31]. In 2002, Newbury-Birch explained the reasons that fear of making mistakes and workload causes stress in dentists’ initial years of practice [32].

Although, the mean score of stress appeared marginally lower for specialists than non-specialists in our study, the difference in mean score was not significant. Likewise, in a Romanian study, both specialists and non-specialist dentists were equally stressed out [27]. Reduced stress level amongst specialist dentists in the current study possibly explains that the specialist training and post-graduation promote to a higher rank job with increased pay, thereby contributing to stress reduction.

According to the current study, working at a junior level is associated with stress. House officers and post-graduate trainees exhibit higher stress levels than Assistant Professor, Associate Professors, and Professors. These results are in agreement with a study by Alkindi, in which senior dental specialists were less mentally stressed than post-graduate trainees [19]. Thus, it leads to an evidence that senior dentists experience less stress due to their postgraduate education, since it qualifies them for a higher designation and a higher salary. In addition to that, the dual role of teaching and clinical practice has been previously considered a means of reducing work-related stress in dentists [33].

The authors believe that the present study is the first one in Pakistan to investigate a novel aspect of reduced stress related to age and professional standing. Although, the area is noteworthy and requires dental professionals' input to discuss the issue of stress in dentistry, hesitation in participation in the study, mainly from senior and older dentists has been observed by the researcher. One of the main limitations of the present study recognised by the authors is a small sample size, particularly low numbers of older participants, which has restricted the authors from improving the generalisation of the study. An additional file of complete data set shows small numbers of senior dentists [see Additional file 2]. Few studies on Pakistani dentists were available to compare the results. However, this supports the authors’ claim that the current study is a new one in Pakistan which has explored the association of stress with demographic and professional backgrounds.

## Conclusions

High stress is common among young and junior dentists in Karachi. Dentists with senior status reported lower stress in the study. More studies are required to assess further association of stress with work amongst the dentists of Pakistan on a larger scale. Exploratory studies are required to discover an effective coping strategy to deal with occupational stress among young and inexperienced dentists in Karachi.

## Supplementary Information

**Additional file 1. **Frequency of all variables and PSS scores.

**Additional file 2. **Complete data set.

## Data Availability

The data-sets generated and analysed during this study are included in this published article and its supplementary information files.

## Abbreviations

PSS: Perceived stress score
Asst., AP: Assistant Professor
Asso.: Associate Professor
Prof.: Professor
PKR: Pakistani rupees
GDPs: General dental practitioners
