# Supplementary material for: Levels of perceived stress according to professional standings among dental surgeons of Karachi: a descriptive study
Source: BMC Oral Health. 2022 Jun 18;22:243. doi: 10.1186/s12903-022-02272-5 (PMC9206731; doi:10.1186/s12903-022-02272-5)
Supplement: Supplementary file 1 — Additional file 1. Frequency of all variables and PSS scores. [file 12903_2022_2272_MOESM1_ESM.docx]

**File:3 Data output for stress study.**

**Note: abbreviation/codes; NDP=NON-DENTAL POSTGRADUATION,**

**Assistant and Asso. Prof= ASSISTANT PROFESSOR AND ASSOCIATE PROFESSOR,**

**PROF =PROFESSOR,**

**ASST.P =ASSISTANT PROFESOR,**

**ASSO.P =ASSOCIATE PROFESSOR**

**HO = HOUSE OFFICERS**

**PG = POST-GRAD TRAINEE**

**DP = DENTAL PRACTITIONER**

**L/LEC = LECTURER**

**Frequencies**

| **Statistics** | | | | | | | | | |
| --- | --- | --- | --- | --- | --- | --- | --- | --- | --- |
|  | | gender2 | ms2 | design2 | WS2 | MINC2 | speciality2 | age_cat | YOE_cat |
| N | Valid | 157 | 157 | 157 | 157 | 157 | 157 | 157 | 156 |
|  | Missing | 0 | 0 | 0 | 0 | 0 | 0 | 0 | 1 |

| **Statistics** | | |
| --- | --- | --- |
|  | | PSS_cat |
| N | Valid | 157 |
|  | Missing | 0 |

**Frequency Table**

| **gender2** | | | | | |
| --- | --- | --- | --- | --- | --- |
|  | | Frequency | Percent | Valid Percent | Cumulative Percent |
| Valid | male | 55 | 35.0 | 35.0 | 35.0 |
|  | female | 102 | 65.0 | 65.0 | 100.0 |
|  | Total | 157 | 100.0 | 100.0 |  |

| **ms2** | | | | | |
| --- | --- | --- | --- | --- | --- |
|  | | Frequency | Percent | Valid Percent | Cumulative Percent |
| Valid | married | 57 | 36.3 | 36.3 | 36.3 |
|  | single | 100 | 63.7 | 63.7 | 100.0 |
|  | Total | 157 | 100.0 | 100.0 |  |

| **design2** | | | | | |
| --- | --- | --- | --- | --- | --- |
|  | | Frequency | Percent | Valid Percent | Cumulative Percent |
| Valid | house officers | 60 | 38.2 | 38.2 | 38.2 |
|  | PG | 24 | 15.3 | 15.3 | 53.5 |
|  | Lecturer | 30 | 19.1 | 19.1 | 72.6 |
|  | Assistant and Asso. Prof | 11 | 7.0 | 7.0 | 79.6 |
|  | dental practitioner | 27 | 17.2 | 17.2 | 96.8 |
|  | prof | 5 | 3.2 | 3.2 | 100.0 |
|  | Total | 157 | 100.0 | 100.0 |  |

| **WS2** | | | | | |
| --- | --- | --- | --- | --- | --- |
|  | | Frequency | Percent | Valid Percent | Cumulative Percent |
| Valid | govt | 36 | 22.9 | 22.9 | 22.9 |
|  | private | 121 | 77.1 | 77.1 | 100.0 |
|  | Total | 157 | 100.0 | 100.0 |  |

| **MINC2** | | | | | |
| --- | --- | --- | --- | --- | --- |
|  | | Frequency | Percent | Valid Percent | Cumulative Percent |
| Valid | honoree | 9 | 5.7 | 5.7 | 5.7 |
|  | 5+ | 42 | 26.8 | 26.8 | 32.5 |
|  | 20+ | 66 | 42.0 | 42.0 | 74.5 |
|  | 60+ | 22 | 14.0 | 14.0 | 88.5 |
|  | 1lac+ | 18 | 11.5 | 11.5 | 100.0 |
|  | Total | 157 | 100.0 | 100.0 |  |

| **speciality2** | | | | | |
| --- | --- | --- | --- | --- | --- |
|  | | Frequency | Percent | Valid Percent | Cumulative Percent |
| Valid | NDP (nondental postgraduation) | 9 | 5.7 | 5.7 | 5.7 |
|  | general dentist | 81 | 51.6 | 51.6 | 57.3 |
|  | all others | 67 | 42.7 | 42.7 | 100.0 |
|  | Total | 157 | 100.0 | 100.0 |  |

| **age_cat** | | | | | |
| --- | --- | --- | --- | --- | --- |
|  | | Frequency | Percent | Valid Percent | Cumulative Percent |
| Valid | 20-29years | 115 | 73.2 | 73.2 | 73.2 |
|  | 30-39years | 29 | 18.5 | 18.5 | 91.7 |
|  | 40-49years | 7 | 4.5 | 4.5 | 96.2 |
|  | 50 above | 6 | 3.8 | 3.8 | 100.0 |
|  | Total | 157 | 100.0 | 100.0 |  |

| **YOE_cat** | | | | | |
| --- | --- | --- | --- | --- | --- |
|  | | Frequency | Percent | Valid Percent | Cumulative Percent |
| Valid | upto 5 years | 92 | 58.6 | 59.0 | 59.0 |
|  | 6-10 | 29 | 18.5 | 18.6 | 77.6 |
|  | 11 above | 35 | 22.3 | 22.4 | 100.0 |
|  | Total | 156 | 99.4 | 100.0 |  |
| Missing | System | 1 | .6 |  |  |
| Total | | 157 | 100.0 |  |  |

| **PSS_cat** | | | | | |
| --- | --- | --- | --- | --- | --- |
|  | | Frequency | Percent | Valid Percent | Cumulative Percent |
| Valid | low stress | 22 | 14.0 | 14.0 | 14.0 |
|  | moderate stress | 65 | 41.4 | 41.4 | 55.4 |
|  | high stress | 70 | 44.6 | 44.6 | 100.0 |
|  | Total | 157 | 100.0 | 100.0 |  |

SORT CASES BY YOE (A).

SORT CASES BY YOE (D).

**Frequencies**

| **Statistics** | | |
| --- | --- | --- |
| YOE_cat | | |
| N | Valid | 157 |
|  | Missing | 0 |

| **YOE_cat** | | | | | |
| --- | --- | --- | --- | --- | --- |
|  | | Frequency | Percent | Valid Percent | Cumulative Percent |
| Valid | upto 5 years | 92 | 58.6 | 58.6 | 58.6 |
|  | 6-10 | 29 | 18.5 | 18.5 | 77.1 |
|  | 11 above | 36 | 22.9 | 22.9 | 100.0 |
|  | Total | 157 | 100.0 | 100.0 |  |

FREQUENCIES VARIABLES=AGE YOE PSS

/FORMAT=NOTABLE

/STATISTICS=STDDEV MINIMUM MAXIMUM MEAN MEDIAN MODE

/ORDER=ANALYSIS.

**Frequencies**

| **Statistics** | | | | |
| --- | --- | --- | --- | --- |
|  | | AGE | YOE | PSS |
| N | Valid | 157 | 157 | 157 |
|  | Missing | 0 | 0 | 0 |
| Mean | | 28.61 | 7.41 | 18.35 |
| Median | | 26.00 | 4.00 | 18.00 |
| Mode | | 23^a^ | 1 | 16^a^ |
| Std. Deviation | | 7.516 | 8.058 | 5.417 |
| Minimum | | 20 | 1 | 2 |
| Maximum | | 65 | 42 | 35 |

| a. Multiple modes exist. The smallest value is shown |
| --- |

NPAR TESTS

/K-S(NORMAL)=AGE YOE PSS

/MISSING ANALYSIS.

**NPar Tests**

| a. Based on availability of workspace memory. |
| --- |

| **One-Sample Kolmogorov-Smirnov Test** | | | | |
| --- | --- | --- | --- | --- |
|  | | AGE | YOE | PSS |
| N | | 157 | 157 | 157 |
| Normal Parameters^a,b^ | Mean | 28.61 | 7.41 | 18.35 |
|  | Std. Deviation | 7.516 | 8.058 | 5.417 |
| Most Extreme Differences | Absolute | .221 | .238 | .083 |
|  | Positive | .221 | .238 | .083 |
|  | Negative | -.177 | -.213 | -.071 |
| Kolmogorov-Smirnov Z | | 2.775 | 2.984 | 1.041 |
| Asymp. Sig. (2-tailed) | | .000 | .000 | .229 |

| a. Test distribution is Normal. |
| --- |
| b. Calculated from data. |

T-TEST GROUPS=gender2(1 2)

/MISSING=ANALYSIS

/VARIABLES=PSS

/CRITERIA=CI(.95).

**T-Test**

| **Group Statistics** | | | | | |
| --- | --- | --- | --- | --- | --- |
|  | gender2 | N | Mean | Std. Deviation | Std. Error Mean |
| PSS | male | 55 | 17.42 | 4.425 | .597 |
|  | female | 102 | 18.85 | 5.842 | .578 |

| **Independent Samples Test** | | | | | | |
| --- | --- | --- | --- | --- | --- | --- |
|  | | Levene's Test for Equality of Variances | | t-test for Equality of Means | | |
|  |  | F | Sig. | t | df | Sig. (2-tailed) |
|  |  |  |  |  |  |  |
| PSS | Equal variances assumed | 5.466 | .021 | -1.591 | 155 | .114 |
|  | Equal variances not assumed |  |  | -1.727 | 138.016 | .086 |

| **Independent Samples Test** | | | | | |
| --- | --- | --- | --- | --- | --- |
|  | | t-test for Equality of Means | | | |
|  |  | Mean Difference | Std. Error Difference | 95% Confidence Interval of the Difference | |
|  |  |  |  | Lower | Upper |
| PSS | Equal variances assumed | -1.435 | .902 | -3.216 | .347 |
|  | Equal variances not assumed | -1.435 | .831 | -3.078 | .208 |

**T-Test**

| **Group Statistics** | | | | | |
| --- | --- | --- | --- | --- | --- |
|  | ms2 | N | Mean | Std. Deviation | Std. Error Mean |
| PSS | married | 57 | 16.72 | 5.421 | .718 |
|  | single | 100 | 19.28 | 5.217 | .522 |

| **Independent Samples Test** | | | | | | |
| --- | --- | --- | --- | --- | --- | --- |
|  | | Levene's Test for Equality of Variances | | t-test for Equality of Means | | |
|  |  | F | Sig. | t | df | Sig. (2-tailed) |
|  |  |  |  |  |  |  |
| PSS | Equal variances assumed | .313 | .577 | -2.916 | 155 | .004 |
|  | Equal variances not assumed |  |  | -2.885 | 112.929 | .005 |

| **Independent Samples Test** | | | | | |
| --- | --- | --- | --- | --- | --- |
|  | | t-test for Equality of Means | | | |
|  |  | Mean Difference | Std. Error Difference | 95% Confidence Interval of the Difference | |
|  |  |  |  | Lower | Upper |
| PSS | Equal variances assumed | -2.561 | .878 | -4.295 | -.826 |
|  | Equal variances not assumed | -2.561 | .887 | -4.319 | -.802 |

**T-Test**

| **Group Statistics** | | | | | |
| --- | --- | --- | --- | --- | --- |
|  | WS2 | N | Mean | Std. Deviation | Std. Error Mean |
| PSS | govt | 36 | 20.39 | 4.692 | .782 |
|  | private | 121 | 17.74 | 5.487 | .499 |

| **Independent Samples Test** | | | | | | | |
| --- | --- | --- | --- | --- | --- | --- | --- |
|  | | Levene's Test for Equality of Variances | | t-test for Equality of Means | | | |
|  |  | F | Sig. | t | df | Sig. (2-tailed) | Mean Difference |
|  |  |  |  |  |  |  |  |
| PSS | Equal variances assumed | 1.200 | .275 | 2.620 | 155 | .010 | 2.645 |
|  | Equal variances not assumed |  |  | 2.852 | 66.086 | .006 | 2.645 |

| **Independent Samples Test** | | | | |
| --- | --- | --- | --- | --- |
|  | | t-test for Equality of Means | | |
|  |  | Std. Error Difference | 95% Confidence Interval of the Difference | |
|  |  |  | Lower | Upper |
| PSS | Equal variances assumed | 1.010 | .651 | 4.639 |
|  | Equal variances not assumed | .928 | .793 | 4.497 |

**Oneway by designation(design2):**

| **Descriptives** | | | | | | | |
| --- | --- | --- | --- | --- | --- | --- | --- |
| PSS | | | | | | | |
|  | N | Mean | Std. Deviation | Std. Error | 95% Confidence Interval for Mean | | Minimum |
|  |  |  |  |  | Lower Bound | Upper Bound |  |
| house officers | 60 | 18.93 | 5.099 | .658 | 17.62 | 20.25 | 10 |
| PG | 24 | 18.67 | 4.724 | .964 | 16.67 | 20.66 | 6 |
| Lecturer | 30 | 17.53 | 5.270 | .962 | 15.57 | 19.50 | 6 |
| Assistant and Asso. Prof. | 11 | 15.45 | 5.646 | 1.702 | 11.66 | 19.25 | 8 |
| dental practitioner | 27 | 20.11 | 5.169 | .995 | 18.07 | 22.16 | 9 |
| prof | 5 | 11.60 | 8.142 | 3.641 | 1.49 | 21.71 | 2 |
| Total | 157 | 18.35 | 5.417 | .432 | 17.50 | 19.20 | 2 |

| **Descriptives** | |
| --- | --- |
| PSS | |
|  | Maximum |
|  |  |
| house officers | 35 |
| PG | 28 |
| Lecturer | 30 |
| Assistant and Asso. Prof. | 25 |
| dental practitioner | 30 |
| prof | 23 |
| Total | 35 |

| **Test of Homogeneity of Variances** | | | |
| --- | --- | --- | --- |
| PSS | | | |
| Levene Statistic | df1 | df2 | Sig. |
| .697 | 5 | 151 | .626 |

| **ANOVA** | | | | | |
| --- | --- | --- | --- | --- | --- |
| PSS | | | | | |
|  | Sum of Squares | df | Mean Square | F | Sig. |
| Between Groups | 446.605 | 5 | 89.321 | 3.265 | .008 |
| Within Groups | 4131.127 | 151 | 27.358 |  |  |
| Total | 4577.732 | 156 |  |  |  |

**Post Hoc Tests**

| **Multiple Comparisons** | | | | | | |
| --- | --- | --- | --- | --- | --- | --- |
| Dependent Variable: PSS | | | | | | |
| Tukey HSD | | | | | | |
| (I) design2 | (J) design2 | Mean Difference (I-J) | Std. Error | Sig. | 95% Confidence Interval | |
|  |  |  |  |  | Lower Bound | Upper Bound |
| house officers | PG | .267 | 1.263 | 1.000 | -3.38 | 3.91 |
|  | Lecturer | 1.400 | 1.170 | .838 | -1.98 | 4.78 |
|  | Assistant and Asso. Prof. | 3.479 | 1.716 | .332 | -1.47 | 8.43 |
|  | dental practitioner | -1.178 | 1.212 | .926 | -4.68 | 2.32 |
|  | prof | 7.333^*^ | 2.435 | .035 | .31 | 14.36 |
| PG | house officers | -.267 | 1.263 | 1.000 | -3.91 | 3.38 |
|  | Lecturer | 1.133 | 1.432 | .969 | -3.00 | 5.27 |
|  | Assistant and Asso. Prof. | 3.212 | 1.904 | .543 | -2.29 | 8.71 |
|  | dental practitioner | -1.444 | 1.467 | .922 | -5.68 | 2.79 |
|  | prof | 7.067 | 2.571 | .072 | -.36 | 14.49 |
| Lecturer | house officers | -1.400 | 1.170 | .838 | -4.78 | 1.98 |
|  | PG | -1.133 | 1.432 | .969 | -5.27 | 3.00 |
|  | Assistant and Asso. Prof. | 2.079 | 1.844 | .869 | -3.24 | 7.40 |
|  | dental practitioner | -2.578 | 1.388 | .432 | -6.58 | 1.43 |
|  | prof | 5.933 | 2.527 | .182 | -1.36 | 13.23 |
| Assistant and Asso. Prof | house officers | -3.479 | 1.716 | .332 | -8.43 | 1.47 |
|  | PG | -3.212 | 1.904 | .543 | -8.71 | 2.29 |
|  | Lecturer | -2.079 | 1.844 | .869 | -7.40 | 3.24 |
|  | dental practitioner | -4.657 | 1.871 | .134 | -10.06 | .74 |
|  | prof | 3.855 | 2.821 | .747 | -4.29 | 12.00 |
| dental practitioner | house officers | 1.178 | 1.212 | .926 | -2.32 | 4.68 |
|  | PG | 1.444 | 1.467 | .922 | -2.79 | 5.68 |
|  | Lecturer | 2.578 | 1.388 | .432 | -1.43 | 6.58 |
|  | Assistant and Asso. Prof. | 4.657 | 1.871 | .134 | -.74 | 10.06 |
|  | prof | 8.511^*^ | 2.547 | .013 | 1.16 | 15.86 |
| prof | house officers | -7.333^*^ | 2.435 | .035 | -14.36 | -.31 |
|  | PG | -7.067 | 2.571 | .072 | -14.49 | .36 |
|  | Lecturer | -5.933 | 2.527 | .182 | -13.23 | 1.36 |
|  | Assistant and Asso. Prof. | -3.855 | 2.821 | .747 | -12.00 | 4.29 |
|  | dental practitioner | -8.511^*^ | 2.547 | .013 | -15.86 | -1.16 |

| *. The mean difference is significant at the 0.05 level. |
| --- |

**Homogeneous Subsets**

| **PSS** | | | |
| --- | --- | --- | --- |
| Tukey HSD^a,b^ | | | |
| design2 | N | Subset for alpha = 0.05 | |
|  |  | 1 | 2 |
| prof | 5 | 11.60 |  |
| Assistant and Asso. Prof | 11 | 15.45 | 15.45 |
| Lecturer | 30 |  | 17.53 |
| PG | 24 |  | 18.67 |
| house officers | 60 |  | 18.93 |
| dental practitioner | 27 |  | 20.11 |
| Sig. |  | .364 | .170 |

| Means for groups in homogeneous subsets are displayed. |
| --- |
| a. Uses Harmonic Mean Sample Size = 14.299. |
| b. The group sizes are unequal. The harmonic mean of the group sizes is used. Type I error levels are not guaranteed. |

**ONEWAY PSS BY MINC2:**

/STATISTICS DESCRIPTIVES HOMOGENEITY

/MISSING ANALYSIS

/POSTHOC=TUKEY ALPHA(0.05).

**Oneway**

| **Descriptives** | | | | | | | | |
| --- | --- | --- | --- | --- | --- | --- | --- | --- |
| PSS | | | | | | | | |
|  | N | Mean | Std. Deviation | Std. Error | 95% Confidence Interval for Mean | | Minimum | Maximum |
|  |  |  |  |  | Lower Bound | Upper Bound |  |  |
| honoree | 9 | 21.67 | 6.910 | 2.303 | 16.36 | 26.98 | 11 | 35 |
| 5+ | 42 | 19.29 | 4.539 | .700 | 17.87 | 20.70 | 10 | 30 |
| 20+ | 66 | 18.12 | 5.003 | .616 | 16.89 | 19.35 | 6 | 30 |
| 60+ | 22 | 18.73 | 4.891 | 1.043 | 16.56 | 20.90 | 6 | 30 |
| 1lac+ | 18 | 14.89 | 7.128 | 1.680 | 11.34 | 18.43 | 2 | 26 |
| Total | 157 | 18.35 | 5.417 | .432 | 17.50 | 19.20 | 2 | 35 |

| **Test of Homogeneity of Variances** | | | |
| --- | --- | --- | --- |
| PSS | | | |
| Levene Statistic | df1 | df2 | Sig. |
| 2.020 | 4 | 152 | .094 |

| **ANOVA** | | | | | |
| --- | --- | --- | --- | --- | --- |
| PSS | | | | | |
|  | Sum of Squares | df | Mean Square | F | Sig. |
| Between Groups | 357.989 | 4 | 89.497 | 3.224 | .014 |
| Within Groups | 4219.743 | 152 | 27.761 |  |  |
| Total | 4577.732 | 156 |  |  |  |

**Post Hoc Tests**

| **Multiple Comparisons** | | | | | | |
| --- | --- | --- | --- | --- | --- | --- |
| Dependent Variable: PSS | | | | | | |
| Tukey HSD | | | | | | |
| (I) MINC2 | (J) MINC2 | Mean Difference (I-J) | Std. Error | Sig. | 95% Confidence Interval | |
|  |  |  |  |  | Lower Bound | Upper Bound |
| honoree | 5+ | 2.381 | 1.935 | .734 | -2.96 | 7.72 |
|  | 20+ | 3.545 | 1.872 | .325 | -1.62 | 8.71 |
|  | 60+ | 2.939 | 2.085 | .622 | -2.82 | 8.70 |
|  | 1lac+ | 6.778^*^ | 2.151 | .017 | .84 | 12.72 |
| 5+ | honoree | -2.381 | 1.935 | .734 | -7.72 | 2.96 |
|  | 20+ | 1.165 | 1.040 | .796 | -1.71 | 4.04 |
|  | 60+ | .558 | 1.387 | .994 | -3.27 | 4.39 |
|  | 1lac+ | 4.397^*^ | 1.484 | .029 | .30 | 8.49 |
| 20+ | honoree | -3.545 | 1.872 | .325 | -8.71 | 1.62 |
|  | 5+ | -1.165 | 1.040 | .796 | -4.04 | 1.71 |
|  | 60+ | -.606 | 1.297 | .990 | -4.19 | 2.98 |
|  | 1lac+ | 3.232 | 1.401 | .148 | -.64 | 7.10 |
| 60+ | honoree | -2.939 | 2.085 | .622 | -8.70 | 2.82 |
|  | 5+ | -.558 | 1.387 | .994 | -4.39 | 3.27 |
|  | 20+ | .606 | 1.297 | .990 | -2.98 | 4.19 |
|  | 1lac+ | 3.838 | 1.675 | .153 | -.78 | 8.46 |
| 1lac+ | honoree | -6.778^*^ | 2.151 | .017 | -12.72 | -.84 |
|  | 5+ | -4.397^*^ | 1.484 | .029 | -8.49 | -.30 |
|  | 20+ | -3.232 | 1.401 | .148 | -7.10 | .64 |
|  | 60+ | -3.838 | 1.675 | .153 | -8.46 | .78 |

| *. The mean difference is significant at the 0.05 level. |
| --- |

**Homogeneous Subsets**

| **PSS** | | | |
| --- | --- | --- | --- |
| Tukey HSD^a,b^ | | | |
| MINC2 | N | Subset for alpha = 0.05 | |
|  |  | 1 | 2 |
| 1lac+ | 18 | 14.89 |  |
| 20+ | 66 | 18.12 | 18.12 |
| 60+ | 22 | 18.73 | 18.73 |
| 5+ | 42 | 19.29 | 19.29 |
| honoree | 9 |  | 21.67 |
| Sig. |  | .069 | .216 |

| Means for groups in homogeneous subsets are displayed. |
| --- |
| a. Uses Harmonic Mean Sample Size = 19.914. |
| b. The group sizes are unequal. The harmonic mean of the group sizes is used. Type I error levels are not guaranteed. |

**ONEWAY PSS BY speciality2:**

/STATISTICS DESCRIPTIVES HOMOGENEITY

/MISSING ANALYSIS

/POSTHOC=TUKEY ALPHA(0.05).

**Oneway**

| **Descriptives** | | | | | | | | |
| --- | --- | --- | --- | --- | --- | --- | --- | --- |
| PSS | | | | | | | | |
|  | N | Mean | Std. Deviation | Std. Error | 95% Confidence Interval for Mean | | Minimum | Maximum |
|  |  |  |  |  | Lower Bound | Upper Bound |  |  |
| NDP | 9 | 18.56 | 4.773 | 1.591 | 14.89 | 22.22 | 12 | 27 |
| general dentist | 81 | 18.81 | 4.661 | .518 | 17.78 | 19.85 | 9 | 30 |
| all others | 67 | 17.76 | 6.296 | .769 | 16.23 | 19.30 | 2 | 35 |
| Total | 157 | 18.35 | 5.417 | .432 | 17.50 | 19.20 | 2 | 35 |

| **Test of Homogeneity of Variances** | | | |
| --- | --- | --- | --- |
| PSS | | | |
| Levene Statistic | df1 | df2 | Sig. |
| 2.197 | 2 | 154 | .115 |

| **ANOVA** | | | | | |
| --- | --- | --- | --- | --- | --- |
| PSS | | | | | |
|  | Sum of Squares | df | Mean Square | F | Sig. |
| Between Groups | 41.109 | 2 | 20.554 | .698 | .499 |
| Within Groups | 4536.624 | 154 | 29.459 |  |  |
| Total | 4577.732 | 156 |  |  |  |

**Post Hoc Tests**

| **Multiple Comparisons** | | | | | | |
| --- | --- | --- | --- | --- | --- | --- |
| Dependent Variable: PSS | | | | | | |
| Tukey HSD | | | | | | |
| (I) speciality2 | (J) speciality2 | Mean Difference (I-J) | Std. Error | Sig. | 95% Confidence Interval | |
|  |  |  |  |  | Lower Bound | Upper Bound |
| NDP | general dentist | -.259 | 1.907 | .990 | -4.77 | 4.25 |
|  | all others | .794 | 1.927 | .911 | -3.77 | 5.35 |
| general dentist | NDP | .259 | 1.907 | .990 | -4.25 | 4.77 |
|  | all others | 1.054 | .896 | .470 | -1.07 | 3.17 |
| all others | NDP | -.794 | 1.927 | .911 | -5.35 | 3.77 |
|  | general dentist | -1.054 | .896 | .470 | -3.17 | 1.07 |

**Homogeneous Subsets**

| **PSS** | | |
| --- | --- | --- |
| Tukey HSD^a,b^ | | |
| speciality2 | N | Subset for alpha = 0.05 |
|  |  | 1 |
| all others | 67 | 17.76 |
| NDP | 9 | 18.56 |
| general dentist | 81 | 18.81 |
| Sig. |  | .799 |

| Means for groups in homogeneous subsets are displayed. |
| --- |
| a. Uses Harmonic Mean Sample Size = 21.679. |
| b. The group sizes are unequal. The harmonic mean of the group sizes is used. Type I error levels are not guaranteed. |

**ONEWAY PSS BY age_cat**

/STATISTICS DESCRIPTIVES HOMOGENEITY

/MISSING ANALYSIS

/POSTHOC=TUKEY ALPHA(0.05).

**Oneway**

| **Descriptives** | | | | | | | | |
| --- | --- | --- | --- | --- | --- | --- | --- | --- |
| PSS | | | | | | | | |
|  | N | Mean | Std. Deviation | Std. Error | 95% Confidence Interval for Mean | | Minimum | Maximum |
|  |  |  |  |  | Lower Bound | Upper Bound |  |  |
| 20-29years | 115 | 19.01 | 5.428 | .506 | 18.01 | 20.01 | 2 | 35 |
| 30-39years | 29 | 17.55 | 4.702 | .873 | 15.76 | 19.34 | 6 | 25 |
| 40-49years | 7 | 12.86 | 4.981 | 1.883 | 8.25 | 17.46 | 6 | 18 |
| 50 above | 6 | 16.00 | 5.254 | 2.145 | 10.49 | 21.51 | 9 | 24 |
| Total | 157 | 18.35 | 5.417 | .432 | 17.50 | 19.20 | 2 | 35 |

| **Test of Homogeneity of Variances** | | | |
| --- | --- | --- | --- |
| PSS | | | |
| Levene Statistic | df1 | df2 | Sig. |
| .098 | 3 | 153 | .961 |

| **ANOVA** | | | | | |
| --- | --- | --- | --- | --- | --- |
| PSS | | | | | |
|  | Sum of Squares | df | Mean Square | F | Sig. |
| Between Groups | 312.712 | 3 | 104.237 | 3.739 | .013 |
| Within Groups | 4265.021 | 153 | 27.876 |  |  |
| Total | 4577.732 | 156 |  |  |  |

**Post Hoc Tests**

| **Multiple Comparisons** | | | | | | |
| --- | --- | --- | --- | --- | --- | --- |
| Dependent Variable: PSS | | | | | | |
| Tukey HSD | | | | | | |
| (I) age_cat | (J) age_cat | Mean Difference (I-J) | Std. Error | Sig. | 95% Confidence Interval | |
|  |  |  |  |  | Lower Bound | Upper Bound |
| 20-29years | 30-39years | 1.457 | 1.097 | .547 | -1.39 | 4.31 |
|  | 40-49years | 6.152^*^ | 2.055 | .017 | .81 | 11.49 |
|  | 50 above | 3.009 | 2.211 | .526 | -2.73 | 8.75 |
| 30-39years | 20-29years | -1.457 | 1.097 | .547 | -4.31 | 1.39 |
|  | 40-49years | 4.695 | 2.223 | .154 | -1.08 | 10.47 |
|  | 50 above | 1.552 | 2.368 | .914 | -4.60 | 7.70 |
| 40-49years | 20-29years | -6.152^*^ | 2.055 | .017 | -11.49 | -.81 |
|  | 30-39years | -4.695 | 2.223 | .154 | -10.47 | 1.08 |
|  | 50 above | -3.143 | 2.937 | .708 | -10.77 | 4.49 |
| 50 above | 20-29years | -3.009 | 2.211 | .526 | -8.75 | 2.73 |
|  | 30-39years | -1.552 | 2.368 | .914 | -7.70 | 4.60 |
|  | 40-49years | 3.143 | 2.937 | .708 | -4.49 | 10.77 |

| *. The mean difference is significant at the 0.05 level. |
| --- |

**Homogeneous Subsets**

| **PSS** | | | |
| --- | --- | --- | --- |
| Tukey HSD^a,b^ | | | |
| age_cat | N | Subset for alpha = 0.05 | |
|  |  | 1 | 2 |
| 40-49years | 7 | 12.86 |  |
| 50 above | 6 | 16.00 | 16.00 |
| 30-39years | 29 | 17.55 | 17.55 |
| 20-29years | 115 |  | 19.01 |
| Sig. |  | .152 | .528 |

| Means for groups in homogeneous subsets are displayed. |
| --- |
| a. Uses Harmonic Mean Sample Size = 11.341. |
| b. The group sizes are unequal. The harmonic mean of the group sizes is used. Type I error levels are not guaranteed. |

**ONEWAY PSS BY YOE_cat**

/STATISTICS DESCRIPTIVES HOMOGENEITY

/MISSING ANALYSIS

/POSTHOC=TUKEY ALPHA(0.05).

**Oneway**

| **Descriptives** | | | | | | | | |
| --- | --- | --- | --- | --- | --- | --- | --- | --- |
| PSS | | | | | | | | |
|  | N | Mean | Std. Deviation | Std. Error | 95% Confidence Interval for Mean | | Minimum | Maximum |
|  |  |  |  |  | Lower Bound | Upper Bound |  |  |
| upto 5 years | 92 | 19.29 | 5.230 | .545 | 18.21 | 20.38 | 10 | 35 |
| 6-10 | 29 | 18.72 | 4.250 | .789 | 17.11 | 20.34 | 6 | 27 |
| 11-above | 36 | 15.64 | 5.934 | .989 | 13.63 | 17.65 | 2 | 25 |
| Total | 157 | 18.35 | 5.417 | .432 | 17.50 | 19.20 | 2 | 35 |

| **Test of Homogeneity of Variances** | | | |
| --- | --- | --- | --- |
| PSS | | | |
| Levene Statistic | df1 | df2 | Sig. |
| 2.639 | 2 | 154 | .075 |

| **ANOVA** | | | | | |
| --- | --- | --- | --- | --- | --- |
| PSS | | | | | |
|  | Sum of Squares | df | Mean Square | F | Sig. |
| Between Groups | 350.558 | 2 | 175.279 | 6.386 | .002 |
| Within Groups | 4227.175 | 154 | 27.449 |  |  |
| Total | 4577.732 | 156 |  |  |  |

**Post Hoc Tests**

| **Multiple Comparisons** | | | | | | |
| --- | --- | --- | --- | --- | --- | --- |
| Dependent Variable: PSS | | | | | | |
| Tukey HSD | | | | | | |
| (I) YOE_cat | (J) YOE_cat | Mean Difference (I-J) | Std. Error | Sig. | 95% Confidence Interval | |
|  |  |  |  |  | Lower Bound | Upper Bound |
| upto 5 years | 6-10 | .569 | 1.116 | .866 | -2.07 | 3.21 |
|  | 11-above | 3.655^*^ | 1.030 | .001 | 1.22 | 6.09 |
| 6-10 | upto 5 years | -.569 | 1.116 | .866 | -3.21 | 2.07 |
|  | 11-above | 3.085 | 1.307 | .051 | -.01 | 6.18 |
| 11-above | upto 5 years | -3.655^*^ | 1.030 | .001 | -6.09 | -1.22 |
|  | 6-10 | -3.085 | 1.307 | .051 | -6.18 | .01 |

| *. The mean difference is significant at the 0.05 level. |
| --- |

**Homogeneous Subsets**

| **PSS** | | | |
| --- | --- | --- | --- |
| Tukey HSD^a,b^ | | | |
| YOE_cat | N | Subset for alpha = 0.05 | |
|  |  | 1 | 2 |
| 11-above | 36 | 15.64 |  |
| 6-10 | 29 |  | 18.72 |
| upto 5 years | 92 |  | 19.29 |
| Sig. |  | 1.000 | .875 |

| Means for groups in homogeneous subsets are displayed. |
| --- |
| a. Uses Harmonic Mean Sample Size = 41.023. |
| b. The group sizes are unequal. The harmonic mean of the group sizes is used. Type I error levels are not guaranteed. |

**Correlations**

| **Descriptive Statistics** | | | |
| --- | --- | --- | --- |
|  | Mean | Std. Deviation | N |
| PSS | 18.35 | 5.417 | 157 |
| AGE | 28.61 | 7.516 | 157 |

| **Correlations** | | | |
| --- | --- | --- | --- |
|  | | PSS | AGE |
| PSS | Pearson Correlation | 1 | -.197^*^ |
|  | Sig. (2-tailed) |  | .013 |
|  | N | 157 | 157 |
| AGE | Pearson Correlation | -.197^*^ | 1 |
|  | Sig. (2-tailed) | .013 |  |
|  | N | 157 | 157 |

| *. Correlation is significant at the 0.05 level (2-tailed). |
| --- |

**Correlations**

| **Descriptive Statistics** | | | |
| --- | --- | --- | --- |
|  | Mean | Std. Deviation | N |
| PSS | 18.35 | 5.417 | 157 |
| YOE | 7.41 | 8.058 | 157 |

| **Correlations** | | | |
| --- | --- | --- | --- |
|  | | PSS | YOE |
| PSS | Pearson Correlation | 1 | -.310^**^ |
|  | Sig. (2-tailed) |  | .000 |
|  | N | 157 | 157 |
| YOE | Pearson Correlation | -.310^**^ | 1 |
|  | Sig. (2-tailed) | .000 |  |
|  | N | 157 | 157 |

| **. Correlation is significant at the 0.01 level (2-tailed). |
| --- |

**Frequencies**

| **Statistics** | | |
| --- | --- | --- |
| speciality3 | | |
| N | Valid | 157 |
|  | Missing | 0 |

| **spaciality3** | | | | | |
| --- | --- | --- | --- | --- | --- |
|  | | Frequency | Percent | Valid Percent | Cumulative Percent |
| Valid | 1.00 | 90 | 57.3 | 57.3 | 57.3 |
|  | 2.00 | 67 | 42.7 | 42.7 | 100.0 |
|  | Total | 157 | 100.0 | 100.0 |  |

**FREQUENCIES VARIABLES=spaciality3**

/ORDER=ANALYSIS.

**Frequencies**

| **Statistics** | | |
| --- | --- | --- |
| speciality3 | | |
| N | Valid | 157 |
|  | Missing | 0 |

| **spaciality3** | | | | | |
| --- | --- | --- | --- | --- | --- |
|  | | Frequency | Percent | Valid Percent | Cumulative Percent |
| Valid | non specialist | 90 | 57.3 | 57.3 | 57.3 |
|  | specialist | 67 | 42.7 | 42.7 | 100.0 |
|  | Total | 157 | 100.0 | 100.0 |  |

**T-TEST GROUPS=speciality3**:

/MISSING=ANALYSIS

/VARIABLES=PSS

/CRITERIA=CI(.95).

**T-Test**

| **Group Statistics** | | | | | |
| --- | --- | --- | --- | --- | --- |
|  | speciality3 | N | Mean | Std. Deviation | Std. Error Mean |
| PSS | non specialist | 90 | 18.79 | 4.646 | .490 |
|  | specialist | 67 | 17.76 | 6.296 | .769 |

| **Independent Samples Test** | | | | | | |
| --- | --- | --- | --- | --- | --- | --- |
|  | | Levene's Test for Equality of Variances | | t-test for Equality of Means | | |
|  |  | F | Sig. | t | df | Sig. (2-tailed) |
|  |  |  |  |  |  |  |
| PSS | Equal variances assumed | 4.422 | .037 | 1.177 | 155 | .241 |
|  | Equal variances not assumed |  |  | 1.127 | 116.194 | .262 |

| **Independent Samples Test** | | | | | |
| --- | --- | --- | --- | --- | --- |
|  | | t-test for Equality of Means | | | |
|  |  | Mean Difference | Std. Error Difference | 95% Confidence Interval of the Difference | |
|  |  |  |  | Lower | Upper |
| PSS | Equal variances assumed | 1.028 | .873 | -.697 | 2.752 |
|  | Equal variances not assumed | 1.028 | .912 | -.778 | 2.834 |

**Crosstabs**

| **Case Processing Summary** | | | | | | |
| --- | --- | --- | --- | --- | --- | --- |
|  | Cases | | | | | |
|  | Valid | | Missing | | Total | |
|  | N | Percent | N | Percent | N | Percent |
| speciality3 * PSS_cat | 157 | 100.0% | 0 | 0.0% | 157 | 100.0% |

| **spaciality3 * PSS_cat Crosstabulation** | | | | | | |
| --- | --- | --- | --- | --- | --- | --- |
|  | | | PSS_cat | | | Total |
|  |  |  | low stress | moderate stress | high stress |  |
| speciality3 | non specialist | Count | 10 | 39 | 41 | 90 |
|  |  | % within speciality3 | 11.1% | 43.3% | 45.6% | 100.0% |
|  | specialist | Count | 12 | 26 | 29 | 67 |
|  |  | % within speciality3 | 17.9% | 38.8% | 43.3% | 100.0% |
| Total | | Count | 22 | 65 | 70 | 157 |
|  |  | % within speciality3 | 14.0% | 41.4% | 44.6% | 100.0% |

| **Chi-Square Tests** | | | |
| --- | --- | --- | --- |
|  | Value | df | Asymp. Sig. (2-sided) |
| Pearson Chi-Square | 1.502^a^ | 2 | .472 |
| Likelihood Ratio | 1.485 | 2 | .476 |
| Linear-by-Linear Association | .638 | 1 | .425 |
| N of Valid Cases | 157 |  |  |

| a. 0 cells (.0%) have expected count less than 5. The minimum expected count is 9.39. |
| --- |

**Crosstabs**

| **Case Processing Summary** | | | | | | |
| --- | --- | --- | --- | --- | --- | --- |
|  | Cases | | | | | |
|  | Valid | | Missing | | Total | |
|  | N | Percent | N | Percent | N | Percent |
| gender2 * PSS_cat | 157 | 100.0% | 0 | 0.0% | 157 | 100.0% |

| **gender2 * PSS_cat Crosstabulation** | | | | | | |
| --- | --- | --- | --- | --- | --- | --- |
|  | | | PSS_cat | | | Total |
|  |  |  | low stress | moderate stress | high stress |  |
| gender2 | male | Count | 7 | 30 | 18 | 55 |
|  |  | % within gender2 | 12.7% | 54.5% | 32.7% | 100.0% |
|  | female | Count | 15 | 35 | 52 | 102 |
|  |  | % within gender2 | 14.7% | 34.3% | 51.0% | 100.0% |
| Total | | Count | 22 | 65 | 70 | 157 |
|  |  | % within gender2 | 14.0% | 41.4% | 44.6% | 100.0% |

| **Chi-Square Tests** | | | |
| --- | --- | --- | --- |
|  | Value | df | Asymp. Sig. (2-sided) |
| Pearson Chi-Square | 6.303^a^ | 2 | .043 |
| Likelihood Ratio | 6.308 | 2 | .043 |
| Linear-by-Linear Association | 1.909 | 1 | .167 |
| N of Valid Cases | 157 |  |  |

| a. 0 cells (.0%) have expected count less than 5. The minimum expected count is 7.71. |
| --- |

**Crosstabs**

| **Case Processing Summary** | | | | | | |
| --- | --- | --- | --- | --- | --- | --- |
|  | Cases | | | | | |
|  | Valid | | Missing | | Total | |
|  | N | Percent | N | Percent | N | Percent |
| ms2 * PSS_cat | 157 | 100.0% | 0 | 0.0% | 157 | 100.0% |
| design2 * PSS_cat | 157 | 100.0% | 0 | 0.0% | 157 | 100.0% |
| WS2 * PSS_cat | 157 | 100.0% | 0 | 0.0% | 157 | 100.0% |
| MINC2 * PSS_cat | 157 | 100.0% | 0 | 0.0% | 157 | 100.0% |
| age_cat * PSS_cat | 157 | 100.0% | 0 | 0.0% | 157 | 100.0% |
| YOE_cat * PSS_cat | 157 | 100.0% | 0 | 0.0% | 157 | 100.0% |

**ms2 * PSS_cat**

| **Crosstab** | | | | | | |
| --- | --- | --- | --- | --- | --- | --- |
|  | | | PSS_cat | | | Total |
|  |  |  | low stress | moderate stress | high stress |  |
| ms2 | married | Count | 12 | 27 | 18 | 57 |
|  |  | % within ms2 | 21.1% | 47.4% | 31.6% | 100.0% |
|  | single | Count | 10 | 38 | 52 | 100 |
|  |  | % within ms2 | 10.0% | 38.0% | 52.0% | 100.0% |
| Total | | Count | 22 | 65 | 70 | 157 |
|  |  | % within ms2 | 14.0% | 41.4% | 44.6% | 100.0% |

| **Chi-Square Tests** | | | |
| --- | --- | --- | --- |
|  | Value | df | Asymp. Sig. (2-sided) |
| Pearson Chi-Square | 7.330^a^ | 2 | .026 |
| Likelihood Ratio | 7.358 | 2 | .025 |
| Linear-by-Linear Association | 7.256 | 1 | .007 |
| N of Valid Cases | 157 |  |  |

| a. 0 cells (.0%) have expected count less than 5. The minimum expected count is 7.99. |
| --- |

**design2 * PSS_cat**

| **Crosstab** | | | | | | |
| --- | --- | --- | --- | --- | --- | --- |
|  | | | PSS_cat | | | Total |
|  |  |  | low stress | moderate stress | high stress |  |
| design2 | house officers | Count | 8 | 23 | 29 | 60 |
|  |  | % within design2 | 13.3% | 38.3% | 48.3% | 100.0% |
|  | PG | Count | 1 | 11 | 12 | 24 |
|  |  | % within design2 | 4.2% | 45.8% | 50.0% | 100.0% |
|  | Lecturer | Count | 6 | 13 | 11 | 30 |
|  |  | % within design2 | 20.0% | 43.3% | 36.7% | 100.0% |
|  | Assistant and Asso. Prof | Count | 3 | 5 | 3 | 11 |
|  |  | % within design2 | 27.3% | 45.5% | 27.3% | 100.0% |
|  | dental practitioner | Count | 1 | 12 | 14 | 27 |
|  |  | % within design2 | 3.7% | 44.4% | 51.9% | 100.0% |
|  | prof | Count | 3 | 1 | 1 | 5 |
|  |  | % within design2 | 60.0% | 20.0% | 20.0% | 100.0% |
| Total | | Count | 22 | 65 | 70 | 157 |
|  |  | % within design2 | 14.0% | 41.4% | 44.6% | 100.0% |

| **Chi-Square Tests** | | | |
| --- | --- | --- | --- |
|  | Value | df | Asymp. Sig. (2-sided) |
| Pearson Chi-Square | 16.862^a^ | 10 | .077 |
| Likelihood Ratio | 14.975 | 10 | .133 |
| Linear-by-Linear Association | .906 | 1 | .341 |
| N of Valid Cases | 157 |  |  |

| a. 9 cells (50.0%) have expected count less than 5. The minimum expected count is .70. |
| --- |

**WS2 * PSS_cat**

| **Crosstab** | | | | | | |
| --- | --- | --- | --- | --- | --- | --- |
|  | | | PSS_cat | | | Total |
|  |  |  | low stress | moderate stress | high stress |  |
| WS2 | govt | Count | 2 | 11 | 23 | 36 |
|  |  | % within WS2 | 5.6% | 30.6% | 63.9% | 100.0% |
|  | private | Count | 20 | 54 | 47 | 121 |
|  |  | % within WS2 | 16.5% | 44.6% | 38.8% | 100.0% |
| Total | | Count | 22 | 65 | 70 | 157 |
|  |  | % within WS2 | 14.0% | 41.4% | 44.6% | 100.0% |

| **Chi-Square Tests** | | | |
| --- | --- | --- | --- |
|  | Value | df | Asymp. Sig. (2-sided) |
| Pearson Chi-Square | 7.615^a^ | 2 | .022 |
| Likelihood Ratio | 7.913 | 2 | .019 |
| Linear-by-Linear Association | 7.262 | 1 | .007 |
| N of Valid Cases | 157 |  |  |

| a. 0 cells (.0%) have expected count less than 5. The minimum expected count is 5.04. |
| --- |

**MINC2 * PSS_cat**

| **Crosstab** | | | | | | |
| --- | --- | --- | --- | --- | --- | --- |
|  | | | PSS_cat | | | Total |
|  |  |  | low stress | moderate stress | high stress |  |
| MINC2 | honoree | Count | 1 | 2 | 6 | 9 |
|  |  | % within MINC2 | 11.1% | 22.2% | 66.7% | 100.0% |
|  | 5+ | Count | 3 | 17 | 22 | 42 |
|  |  | % within MINC2 | 7.1% | 40.5% | 52.4% | 100.0% |
|  | 20+ | Count | 9 | 32 | 25 | 66 |
|  |  | % within MINC2 | 13.6% | 48.5% | 37.9% | 100.0% |
|  | 60+ | Count | 2 | 8 | 12 | 22 |
|  |  | % within MINC2 | 9.1% | 36.4% | 54.5% | 100.0% |
|  | 1lac+ | Count | 7 | 6 | 5 | 18 |
|  |  | % within MINC2 | 38.9% | 33.3% | 27.8% | 100.0% |
| Total | | Count | 22 | 65 | 70 | 157 |
|  |  | % within MINC2 | 14.0% | 41.4% | 44.6% | 100.0% |

| **Chi-Square Tests** | | | |
| --- | --- | --- | --- |
|  | Value | df | Asymp. Sig. (2-sided) |
| Pearson Chi-Square | 15.683^a^ | 8 | .047 |
| Likelihood Ratio | 13.609 | 8 | .093 |
| Linear-by-Linear Association | 5.880 | 1 | .015 |
| N of Valid Cases | 157 |  |  |

| a. 5 cells (33.3%) have expected count less than 5. The minimum expected count is 1.26. |
| --- |

**age_cat * PSS_cat**

| **Crosstab** | | | | | | |
| --- | --- | --- | --- | --- | --- | --- |
|  | | | PSS_cat | | | Total |
|  |  |  | low stress | moderate stress | high stress |  |
| age_cat | 20-29years | Count | 13 | 47 | 55 | 115 |
|  |  | % within age_cat | 11.3% | 40.9% | 47.8% | 100.0% |
|  | 30-39years | Count | 4 | 11 | 14 | 29 |
|  |  | % within age_cat | 13.8% | 37.9% | 48.3% | 100.0% |
|  | 40-49years | Count | 3 | 4 | 0 | 7 |
|  |  | % within age_cat | 42.9% | 57.1% | 0.0% | 100.0% |
|  | 50 above | Count | 2 | 3 | 1 | 6 |
|  |  | % within age_cat | 33.3% | 50.0% | 16.7% | 100.0% |
| Total | | Count | 22 | 65 | 70 | 157 |
|  |  | % within age_cat | 14.0% | 41.4% | 44.6% | 100.0% |

| **Chi-Square Tests** | | | |
| --- | --- | --- | --- |
|  | Value | df | Asymp. Sig. (2-sided) |
| Pearson Chi-Square | 11.505^a^ | 6 | .074 |
| Likelihood Ratio | 13.007 | 6 | .043 |
| Linear-by-Linear Association | 7.199 | 1 | .007 |
| N of Valid Cases | 157 |  |  |

| a. 7 cells (58.3%) have expected count less than 5. The minimum expected count is .84. |
| --- |

**YOE_cat * PSS_cat**

| **Crosstab** | | | | | | |
| --- | --- | --- | --- | --- | --- | --- |
|  | | | PSS_cat | | | Total |
|  |  |  | low stress | moderate stress | high stress |  |
| YOE_cat | upto 5 years | Count | 10 | 39 | 43 | 92 |
|  |  | % within YOE_cat | 10.9% | 42.4% | 46.7% | 100.0% |
|  | 6-10 | Count | 1 | 13 | 15 | 29 |
|  |  | % within YOE_cat | 3.4% | 44.8% | 51.7% | 100.0% |
|  | 11-above | Count | 11 | 13 | 12 | 36 |
|  |  | % within YOE_cat | 30.6% | 36.1% | 33.3% | 100.0% |
| Total | | Count | 22 | 65 | 70 | 157 |
|  |  | % within YOE_cat | 14.0% | 41.4% | 44.6% | 100.0% |

| **Chi-Square Tests** | | | |
| --- | --- | --- | --- |
|  | Value | df | Asymp. Sig. (2-sided) |
| Pearson Chi-Square | 11.786^a^ | 4 | .019 |
| Likelihood Ratio | 11.153 | 4 | .025 |
| Linear-by-Linear Association | 4.135 | 1 | .042 |
| N of Valid Cases | 157 |  |  |

| a. 1 cells (11.1%) have expected count less than 5. The minimum expected count is 4.06. |
| --- |

**Frequencies**

| **Statistics** | | |
| --- | --- | --- |
| agecat2 | | |
| N | Valid | 157 |
|  | Missing | 0 |

| **agecat2** | | | | | |
| --- | --- | --- | --- | --- | --- |
|  | | Frequency | Percent | Valid Percent | Cumulative Percent |
| Valid | 1.00 | 115 | 73.2 | 73.2 | 73.2 |
|  | 2.00 | 29 | 18.5 | 18.5 | 91.7 |
|  | 3.00 | 13 | 8.3 | 8.3 | 100.0 |
|  | Total | 157 | 100.0 | 100.0 |  |

FREQUENCIES VARIABLES=agecat2

/ORDER=ANALYSIS.

**Frequencies**

| **Statistics** | | |
| --- | --- | --- |
| agecat2 | | |
| N | Valid | 157 |
|  | Missing | 0 |

| **agecat2** | | | | | |
| --- | --- | --- | --- | --- | --- |
|  | | Frequency | Percent | Valid Percent | Cumulative Percent |
| Valid | 20-29years | 115 | 73.2 | 73.2 | 73.2 |
|  | 30-39years | 29 | 18.5 | 18.5 | 91.7 |
|  | 40 above | 13 | 8.3 | 8.3 | 100.0 |
|  | Total | 157 | 100.0 | 100.0 |  |

**Crosstabs**

| **Case Processing Summary** | | | | | | |
| --- | --- | --- | --- | --- | --- | --- |
|  | Cases | | | | | |
|  | Valid | | Missing | | Total | |
|  | N | Percent | N | Percent | N | Percent |
| agecat2 * PSS_cat | 157 | 100.0% | 0 | 0.0% | 157 | 100.0% |

| **agecat2 * PSS_cat Crosstabulation** | | | | | | |
| --- | --- | --- | --- | --- | --- | --- |
|  | | | PSS_cat | | | Total |
|  |  |  | low stress | moderate stress | high stress |  |
| agecat2 | 20-29years | Count | 13 | 47 | 55 | 115 |
|  |  | % within agecat2 | 11.3% | 40.9% | 47.8% | 100.0% |
|  | 30-39years | Count | 4 | 11 | 14 | 29 |
|  |  | % within agecat2 | 13.8% | 37.9% | 48.3% | 100.0% |
|  | 40 above | Count | 5 | 7 | 1 | 13 |
|  |  | % within agecat2 | 38.5% | 53.8% | 7.7% | 100.0% |
| Total | | Count | 22 | 65 | 70 | 157 |
|  |  | % within agecat2 | 14.0% | 41.4% | 44.6% | 100.0% |

| **Chi-Square Tests** | | | |
| --- | --- | --- | --- |
|  | Value | df | Asymp. Sig. (2-sided) |
| Pearson Chi-Square | 11.055^a^ | 4 | .026 |
| Likelihood Ratio | 11.353 | 4 | .023 |
| Linear-by-Linear Association | 7.173 | 1 | .007 |
| N of Valid Cases | 157 |  |  |

| a. 2 cells (22.2%) have expected count less than 5. The minimum expected count is 1.82. |
| --- |

| **Risk Estimate** | |
| --- | --- |
|  | Value |
| Odds Ratio for agecat2 (20-29years / 30-39years) | ^a^ |

| a. Risk Estimate statistics cannot be computed. They are only computed for a 2*2 table without empty cells. |
| --- |

RECODE design2 (1=1) (2=2) (3=3) (4=4) (5=3) (6=4) INTO desig3.

EXECUTE.

FREQUENCIES VARIABLES=desig3

/ORDER=ANALYSIS.

**Frequencies**

| **Statistics** | | |
| --- | --- | --- |
| desig3 | | |
| N | Valid | 157 |
|  | Missing | 0 |

| **desig3** | | | | | |
| --- | --- | --- | --- | --- | --- |
|  | | Frequency | Percent | Valid Percent | Cumulative Percent |
| Valid | house officers | 60 | 38.2 | 38.2 | 38.2 |
|  | PG | 24 | 15.3 | 15.3 | 53.5 |
|  | lect and DP | 57 | 36.3 | 36.3 | 89.8 |
|  | AP and above | 16 | 10.2 | 10.2 | 100.0 |
|  | Total | 157 | 100.0 | 100.0 |  |

**Crosstabs**

| **Case Processing Summary** | | | | | | |
| --- | --- | --- | --- | --- | --- | --- |
|  | Cases | | | | | |
|  | Valid | | Missing | | Total | |
|  | N | Percent | N | Percent | N | Percent |
| desig3 * PSS_cat | 157 | 100.0% | 0 | 0.0% | 157 | 100.0% |

| **desig3 * PSS_cat Crosstabulation** | | | | | | |
| --- | --- | --- | --- | --- | --- | --- |
|  | | | PSS_cat | | | Total |
|  |  |  | low stress | moderate stress | high stress |  |
| desig3 | house officers | Count | 8 | 23 | 29 | 60 |
|  |  | % within desig3 | 13.3% | 38.3% | 48.3% | 100.0% |
|  | PG | Count | 1 | 11 | 12 | 24 |
|  |  | % within desig3 | 4.2% | 45.8% | 50.0% | 100.0% |
|  | lect and DP | Count | 7 | 25 | 25 | 57 |
|  |  | % within desig3 | 12.3% | 43.9% | 43.9% | 100.0% |
|  | AP and above | Count | 6 | 6 | 4 | 16 |
|  |  | % within desig3 | 37.5% | 37.5% | 25.0% | 100.0% |
| Total | | Count | 22 | 65 | 70 | 157 |
|  |  | % within desig3 | 14.0% | 41.4% | 44.6% | 100.0% |

| **Chi-Square Tests** | | | |
| --- | --- | --- | --- |
|  | Value | df | Asymp. Sig. (2-sided) |
| Pearson Chi-Square | 10.223^a^ | 6 | .116 |
| Likelihood Ratio | 9.118 | 6 | .167 |
| Linear-by-Linear Association | 2.891 | 1 | .089 |
| N of Valid Cases | 157 |  |  |

| a. 2 cells (16.7%) have expected count less than 5. The minimum expected count is 2.24. |
| --- |

| **Risk Estimate** | |
| --- | --- |
|  | Value |
| Odds Ratio for desig3 (house officers / PG) | ^a^ |

| a. Risk Estimate statistics cannot be computed. They are only computed for a 2*2 table without empty cells. |
| --- |

**Crosstabs**

| **Descriptive Statistics** | | | |
| --- | --- | --- | --- |
|  | Mean | Std. Deviation | N |
| PSS | 18.35 | 5.417 | 157 |
| AGE | 28.61 | 7.516 | 157 |

| **Correlations** | | | |
| --- | --- | --- | --- |
|  | | PSS | AGE |
| PSS | Pearson Correlation | 1 | -.197^*^ |
|  | Sig. (2-tailed) |  | .013 |
|  | N | 157 | 157 |
| AGE | Pearson Correlation | -.197^*^ | 1 |
|  | Sig. (2-tailed) | .013 |  |
|  | N | 157 | 157 |

| *. Correlation is significant at the 0.05 level (2-tailed). |
| --- |

CORRELATIONS

/VARIABLES=PSS YOE

/PRINT=TWOTAIL NOSIG

/STATISTICS DESCRIPTIVES

/MISSING=PAIRWISE.

**Correlations**

| **Descriptive Statistics** | | | |
| --- | --- | --- | --- |
|  | Mean | Std. Deviation | N |
| PSS | 18.35 | 5.417 | 157 |
| YOE | 7.41 | 8.058 | 157 |

| **Correlations** | | | |
| --- | --- | --- | --- |
|  | | PSS | YOE |
| PSS | Pearson Correlation | 1 | -.310^**^ |
|  | Sig. (2-tailed) |  | .000 |
|  | N | 157 | 157 |
| YOE | Pearson Correlation | -.310^**^ | 1 |
|  | Sig. (2-tailed) | .000 |  |
|  | N | 157 | 157 |

| **. Correlation is significant at the 0.01 level (2-tailed). |
| --- |

NONPAR CORR

/VARIABLES=PSS YOE

/PRINT=SPEARMAN TWOTAIL NOSIG

/MISSING=PAIRWISE.

**Nonparametric Correlations**

| **Correlations** | | | | |
| --- | --- | --- | --- | --- |
|  | | | PSS | YOE |
| Spearman's rho | PSS | Correlation Coefficient | 1.000 | -.170^*^ |
|  |  | Sig. (2-tailed) | . | .033 |
|  |  | N | 157 | 157 |
|  | YOE | Correlation Coefficient | -.170^*^ | 1.000 |
|  |  | Sig. (2-tailed) | .033 | . |
|  |  | N | 157 | 157 |

| *. Correlation is significant at the 0.05 level (2-tailed). |
| --- |

| **Descriptive Statistics** | | | | | |
| --- | --- | --- | --- | --- | --- |
|  | N | Mean | Std. Deviation | Minimum | Maximum |
| AGE | 157 | 28.61 | 7.516 | 20 | 65 |
| YOE | 157 | 7.41 | 8.058 | 1 | 42 |

| **One-Sample Kolmogorov-Smirnov Test** | | | |
| --- | --- | --- | --- |
|  | | AGE | YOE |
| N | | 157 | 157 |
| Normal Parameters^a,b^ | Mean | 28.61 | 7.41 |
|  | Std. Deviation | 7.516 | 8.058 |
| Most Extreme Differences | Absolute | .221 | .238 |
|  | Positive | .221 | .238 |
|  | Negative | -.177 | -.213 |
| Kolmogorov-Smirnov Z | | 2.775 | 2.984 |
| Asymp. Sig. (2-tailed) | | .000 | .000 |

| a. Test distribution is Normal. |
| --- |
| b. Calculated from data. |

CORRELATIONS

/VARIABLES=PSS AGE

/PRINT=TWOTAIL NOSIG

/STATISTICS DESCRIPTIVES

/MISSING=PAIRWISE.

**Correlations**

| **Descriptive Statistics** | | | |
| --- | --- | --- | --- |
|  | Mean | Std. Deviation | N |
| PSS | 18.35 | 5.417 | 157 |
| AGE | 28.61 | 7.516 | 157 |

| **Correlations** | | | |
| --- | --- | --- | --- |
|  | | PSS | AGE |
| PSS | Pearson Correlation | 1 | -.197^*^ |
|  | Sig. (2-tailed) |  | .013 |
|  | N | 157 | 157 |
| AGE | Pearson Correlation | -.197^*^ | 1 |
|  | Sig. (2-tailed) | .013 |  |
|  | N | 157 | 157 |

| *. Correlation is significant at the 0.05 level (2-tailed). |
| --- |

**Nonparametric Correlations**

| **Correlations** | | | | |
| --- | --- | --- | --- | --- |
|  | | | PSS | AGE |
| Spearman's rho | PSS | Correlation Coefficient | 1.000 | -.133 |
|  |  | Sig. (2-tailed) | . | .096 |
|  |  | N | 157 | 157 |
|  | AGE | Correlation Coefficient | -.133 | 1.000 |
|  |  | Sig. (2-tailed) | .096 | . |
|  |  | N | 157 | 157 |

LOGISTIC REGRESSION VARIABLES PSS

/METHOD=ENTER AGE

/CRITERIA=PIN(.05) POUT(.10) ITERATE(20) CUT(.5).

REGRESSION

/DESCRIPTIVES MEAN STDDEV CORR SIG N

/MISSING LISTWISE

/STATISTICS COEFF OUTS CI(95) R ANOVA

| **Descriptive Statistics** | | | |
| --- | --- | --- | --- |
|  | Mean | Std. Deviation | N |
| PSS | 18.35 | 5.417 | 157 |
| AGE | 28.61 | 7.516 | 157 |

| **Correlations** | | | |
| --- | --- | --- | --- |
|  | | PSS | AGE |
| Pearson Correlation | PSS | 1.000 | -.197 |
|  | AGE | -.197 | 1.000 |
| Sig. (1-tailed) | PSS | . | .007 |
|  | AGE | .007 | . |
| N | PSS | 157 | 157 |
|  | AGE | 157 | 157 |

| **Variables Entered/Removed^a^** | | | |
| --- | --- | --- | --- |
| Model | Variables Entered | Variables Removed | Method |
| 1 | AGE^b^ | . | Enter |

| a. Dependent Variable: PSS |
| --- |
| b. All requested variables entered. |

| **Model Summary** | | | | |
| --- | --- | --- | --- | --- |
| Model | R | R Square | Adjusted R Square | Std. Error of the Estimate |
| 1 | .197^a^ | .039 | .033 | 5.328 |

| a. Predictors: (Constant), AGE |
| --- |

| **ANOVA^a^** | | | | | | |
| --- | --- | --- | --- | --- | --- | --- |
| Model | | Sum of Squares | df | Mean Square | F | Sig. |
| 1 | Regression | 177.364 | 1 | 177.364 | 6.248 | .013^b^ |
|  | Residual | 4400.368 | 155 | 28.389 |  |  |
|  | Total | 4577.732 | 156 |  |  |  |

| a. Dependent Variable: PSS |
| --- |
| b. Predictors: (Constant), AGE |

| **Coefficients^a^** | | | | | | | | |
| --- | --- | --- | --- | --- | --- | --- | --- | --- |
| Model | | Unstandardized Coefficients | | Standardized Coefficients | t | Sig. | 95.0% Confidence Interval for B | |
|  |  | B | Std. Error | Beta |  |  | Lower Bound | Upper Bound |
| 1 | (Constant) | 22.408 | 1.678 |  | 13.352 | .000 | 19.093 | 25.723 |
|  | AGE | -.142 | .057 | -.197 | -2.500 | .013 | -.254 | -.030 |

| a. Dependent Variable: PSS |
| --- |

REGRESSION

/DESCRIPTIVES MEAN STDDEV CORR SIG N

/MISSING LISTWISE

/STATISTICS COEFF OUTS CI(95) R ANOVA

/CRITERIA=PIN(.05) POUT(.10)

/NOORIGIN

/DEPENDENT PSS

/METHOD=ENTER YOE.

| **Descriptive Statistics** | | | |
| --- | --- | --- | --- |
|  | Mean | Std. Deviation | N |
| PSS | 18.35 | 5.417 | 157 |
| YOE | 7.41 | 8.058 | 157 |

| **Correlations** | | | |
| --- | --- | --- | --- |
|  | | PSS | YOE |
| Pearson Correlation | PSS | 1.000 | -.310 |
|  | YOE | -.310 | 1.000 |
| Sig. (1-tailed) | PSS | . | .000 |
|  | YOE | .000 | . |
| N | PSS | 157 | 157 |
|  | YOE | 157 | 157 |

| **Variables Entered/Removed^a^** | | | |
| --- | --- | --- | --- |
| Model | Variables Entered | Variables Removed | Method |
| 1 | YOE^b^ | . | Enter |

| a. Dependent Variable: PSS |
| --- |
| b. All requested variables entered. |

| **Model Summary** | | | | |
| --- | --- | --- | --- | --- |
| Model | R | R Square | Adjusted R Square | Std. Error of the Estimate |
| 1 | .310^a^ | .096 | .090 | 5.167 |

| a. Predictors: (Constant), YOE |
| --- |

| **ANOVA^a^** | | | | | | |
| --- | --- | --- | --- | --- | --- | --- |
| Model | | Sum of Squares | df | Mean Square | F | Sig. |
| 1 | Regression | 439.259 | 1 | 439.259 | 16.452 | .000^b^ |
|  | Residual | 4138.473 | 155 | 26.700 |  |  |
|  | Total | 4577.732 | 156 |  |  |  |

| a. Dependent Variable: PSS |
| --- |
| b. Predictors: (Constant), YOE |

| **Coefficients^a^** | | | | | | | | |
| --- | --- | --- | --- | --- | --- | --- | --- | --- |
| Model | | Unstandardized Coefficients | | Standardized Coefficients | t | Sig. | 95.0% Confidence Interval for B | |
|  |  | B | Std. Error | Beta |  |  | Lower Bound | Upper Bound |
| 1 | (Constant) | 19.893 | .561 |  | 35.461 | .000 | 18.785 | 21.001 |
|  | YOE | -.208 | .051 | -.310 | -4.056 | .000 | -.310 | -.107 |

| a. Dependent Variable: PSS |
| --- |

**Explore**

| **Case Processing Summary** | | | | | | |
| --- | --- | --- | --- | --- | --- | --- |
|  | Cases | | | | | |
|  | Valid | | Missing | | Total | |
|  | N | Percent | N | Percent | N | Percent |
| AGE | 157 | 100.0% | 0 | 0.0% | 157 | 100.0% |
| YOE | 157 | 100.0% | 0 | 0.0% | 157 | 100.0% |
| PSS | 157 | 100.0% | 0 | 0.0% | 157 | 100.0% |

| **Descriptives** | | | | |
| --- | --- | --- | --- | --- |
|  | | | Statistic | Std. Error |
| AGE | Mean | | 28.61 | .600 |
|  | 95% Confidence Interval for Mean | Lower Bound | 27.42 |  |
|  |  | Upper Bound | 29.79 |  |
|  | 5% Trimmed Mean | | 27.63 |  |
|  | Median | | 26.00 |  |
|  | Variance | | 56.497 |  |
|  | Std. Deviation | | 7.516 |  |
|  | Minimum | | 20 |  |
|  | Maximum | | 65 |  |
|  | Range | | 45 |  |
|  | Interquartile Range | | 6 |  |
|  | Skewness | | 2.274 | .194 |
|  | Kurtosis | | 5.868 | .385 |
| YOE | Mean | | 7.41 | .643 |
|  | 95% Confidence Interval for Mean | Lower Bound | 6.14 |  |
|  |  | Upper Bound | 8.68 |  |
|  | 5% Trimmed Mean | | 6.39 |  |
|  | Median | | 4.00 |  |
|  | Variance | | 64.935 |  |
|  | Std. Deviation | | 8.058 |  |
|  | Minimum | | 1 |  |
|  | Maximum | | 42 |  |
|  | Range | | 41 |  |
|  | Interquartile Range | | 8 |  |
|  | Skewness | | 1.875 | .194 |
|  | Kurtosis | | 3.365 | .385 |
| PSS | Mean | | 18.35 | .432 |
|  | 95% Confidence Interval for Mean | Lower Bound | 17.50 |  |
|  |  | Upper Bound | 19.20 |  |
|  | 5% Trimmed Mean | | 18.35 |  |
|  | Median | | 18.00 |  |
|  | Variance | | 29.344 |  |
|  | Std. Deviation | | 5.417 |  |
|  | Minimum | | 2 |  |
|  | Maximum | | 35 |  |
|  | Range | | 33 |  |
|  | Interquartile Range | | 6 |  |
|  | Skewness | | -.014 | .194 |
|  | Kurtosis | | .430 | .385 |

**AGE**

AGE Stem-and-Leaf Plot

Frequency Stem & Leaf

2.00 20 . 00

1.00 21 . 0

5.00 22 . 00000

21.00 23 . 000000000000000000000

17.00 24 . 00000000000000000

21.00 25 . 000000000000000000000

19.00 26 . 0000000000000000000

14.00 27 . 00000000000000

8.00 28 . 00000000

7.00 29 . 0000000

9.00 30 . 000000000

2.00 31 . 00

1.00 32 . 0

1.00 33 . 0

2.00 34 . 00

8.00 35 . 00000000

.00 36 .

4.00 37 . 0000

2.00 38 . 00

13.00 Extremes (>=40.0)

Stem width: 1

Each leaf: 1 case(s)


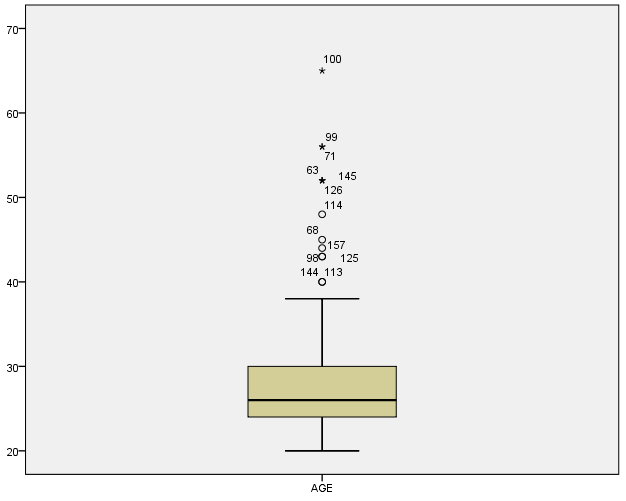


**YOE**

YOE Stem-and-Leaf Plot

Frequency Stem & Leaf

29.00 0 . 11111111111111111111111111111

38.00 0 . 22222222222222222222333333333333333333

25.00 0 . 4444444444444444555555555

16.00 0 . 6666666666666777

9.00 0 . 888999999

6.00 1 . 000011

5.00 1 . 22222

8.00 1 . 44444555

2.00 1 . 67

5.00 1 . 88889

3.00 2 . 111

11.00 Extremes (>=23)

Stem width: 10

Each leaf: 1 case(s)


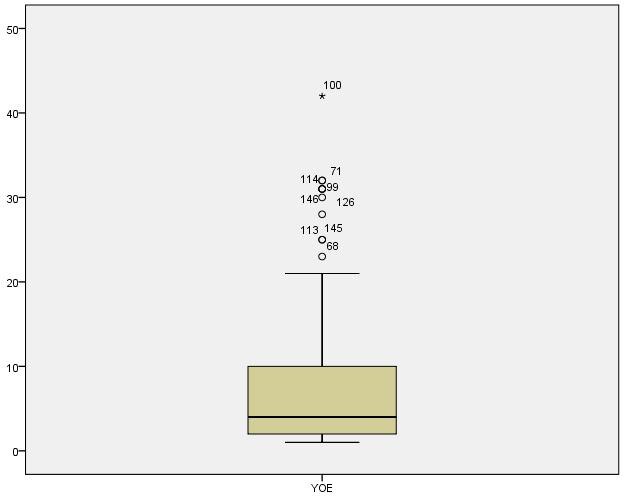


**PSS**

PSS Stem-and-Leaf Plot

Frequency Stem & Leaf

1.00 Extremes (=<2)

.00 0 .

3.00 0 . 666

4.00 0 . 8999

9.00 1 . 000011111

11.00 1 . 22222333333

13.00 1 . 4444455555555

25.00 1 . 6666666666666666677777777

21.00 1 . 888888888888889999999

34.00 2 . 0000000000000000011111111111111111

12.00 2 . 222222233333

9.00 2 . 444444555

8.00 2 . 66777777

2.00 2 . 88

4.00 3 . 0000

1.00 Extremes (>=35)

Stem width: 10

Each leaf: 1 case(s)


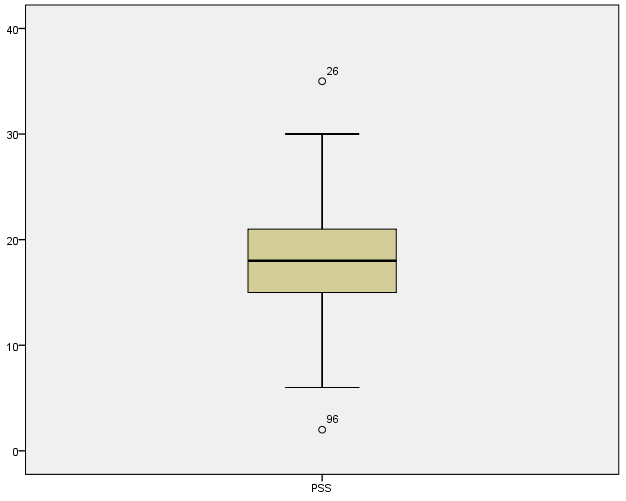


**Descriptives**

| **Descriptive Statistics** | | | | | |
| --- | --- | --- | --- | --- | --- |
|  | N | Minimum | Maximum | Mean | Std. Deviation |
| AGE | 157 | 20 | 65 | 28.61 | 7.516 |
| Valid N (listwise) | 157 |  |  |  |  |

**Frequencies**

| **Statistics** | | |
| --- | --- | --- |
| AGE | | |
| N | Valid | 157 |
|  | Missing | 0 |
| Mean | | 28.61 |
| Std. Deviation | | 7.516 |

| **AGE** | | | | | |
| --- | --- | --- | --- | --- | --- |
|  | | Frequency | Percent | Valid Percent | Cumulative Percent |
| Valid | 20 | 2 | 1.3 | 1.3 | 1.3 |
|  | 21 | 1 | .6 | .6 | 1.9 |
|  | 22 | 5 | 3.2 | 3.2 | 5.1 |
|  | 23 | 21 | 13.4 | 13.4 | 18.5 |
|  | 24 | 17 | 10.8 | 10.8 | 29.3 |
|  | 25 | 21 | 13.4 | 13.4 | 42.7 |
|  | 26 | 19 | 12.1 | 12.1 | 54.8 |
|  | 27 | 14 | 8.9 | 8.9 | 63.7 |
|  | 28 | 8 | 5.1 | 5.1 | 68.8 |
|  | 29 | 7 | 4.5 | 4.5 | 73.2 |
|  | 30 | 9 | 5.7 | 5.7 | 79.0 |
|  | 31 | 2 | 1.3 | 1.3 | 80.3 |
|  | 32 | 1 | .6 | .6 | 80.9 |
|  | 33 | 1 | .6 | .6 | 81.5 |
|  | 34 | 2 | 1.3 | 1.3 | 82.8 |
|  | 35 | 8 | 5.1 | 5.1 | 87.9 |
|  | 37 | 4 | 2.5 | 2.5 | 90.4 |
|  | 38 | 2 | 1.3 | 1.3 | 91.7 |
|  | 40 | 2 | 1.3 | 1.3 | 93.0 |
|  | 43 | 2 | 1.3 | 1.3 | 94.3 |
|  | 44 | 1 | .6 | .6 | 94.9 |
|  | 45 | 1 | .6 | .6 | 95.5 |
|  | 48 | 1 | .6 | .6 | 96.2 |
|  | 52 | 3 | 1.9 | 1.9 | 98.1 |
|  | 56 | 2 | 1.3 | 1.3 | 99.4 |
|  | 65 | 1 | .6 | .6 | 100.0 |
|  | Total | 157 | 100.0 | 100.0 |  |

**DESCRIPTIVES VARIABLES=AGE:**

/STATISTICS=MEAN STDDEV.

**Descriptives**

| **Descriptive Statistics** | | | |
| --- | --- | --- | --- |
|  | N | Mean | Std. Deviation |
| AGE | 157 | 28.61 | 7.516 |
| Valid N (listwise) | 157 |  |  |

MEANS TABLES=AGE BY PSS

/CELLS MEAN COUNT STDDEV.

**Means**

| **Case Processing Summary** | | | | | | |
| --- | --- | --- | --- | --- | --- | --- |
|  | Cases | | | | | |
|  | Included | | Excluded | | Total | |
|  | N | Percent | N | Percent | N | Percent |
| AGE * PSS | 157 | 100.0% | 0 | 0.0% | 157 | 100.0% |

| **Report** | | | |
| --- | --- | --- | --- |
| AGE | | | |
| PSS | Mean | N | Std. Deviation |
| 2 | 25.00 | 1 | . |
| 6 | 33.00 | 3 | 9.849 |
| 8 | 45.00 | 1 | . |
| 9 | 41.33 | 3 | 15.567 |
| 10 | 29.00 | 4 | 5.944 |
| 11 | 26.40 | 5 | 3.050 |
| 12 | 30.20 | 5 | 14.601 |
| 13 | 31.17 | 6 | 5.231 |
| 14 | 27.00 | 5 | 5.050 |
| 15 | 31.62 | 8 | 13.627 |
| 16 | 27.35 | 17 | 7.097 |
| 17 | 30.50 | 8 | 6.655 |
| 18 | 30.50 | 14 | 10.075 |
| 19 | 26.00 | 7 | 3.000 |
| 20 | 27.18 | 17 | 4.334 |
| 21 | 26.65 | 17 | 3.517 |
| 22 | 26.00 | 7 | 4.243 |
| 23 | 32.00 | 5 | 7.778 |
| 24 | 29.83 | 6 | 11.107 |
| 25 | 28.00 | 3 | 7.937 |
| 26 | 26.50 | 2 | .707 |
| 27 | 25.67 | 6 | 1.862 |
| 28 | 25.00 | 2 | 2.828 |
| 30 | 25.75 | 4 | 1.893 |
| 35 | 26.00 | 1 | . |
| Total | 28.61 | 157 | 7.516 |

**MEANS TABLES=PSS BY age_cat:**

/CELLS MEAN COUNT STDDEV.

**Means**

| **Case Processing Summary** | | | | | | |
| --- | --- | --- | --- | --- | --- | --- |
|  | Cases | | | | | |
|  | Included | | Excluded | | Total | |
|  | N | Percent | N | Percent | N | Percent |
| PSS * age_cat | 157 | 100.0% | 0 | 0.0% | 157 | 100.0% |

| **Report** | | | |
| --- | --- | --- | --- |
| PSS | | | |
| age_cat | Mean | N | Std. Deviation |
| 20-29years | 19.01 | 115 | 5.428 |
| 30-39years | 17.55 | 29 | 4.702 |
| 40-49years | 12.86 | 7 | 4.981 |
| 50 above | 16.00 | 6 | 5.254 |
| Total | 18.35 | 157 | 5.417 |

MEANS TABLES=PSS BY agecat2

/CELLS MEAN COUNT STDDEV.

**Means**

| **Case Processing Summary** | | | | | | |
| --- | --- | --- | --- | --- | --- | --- |
|  | Cases | | | | | |
|  | Included | | Excluded | | Total | |
|  | N | Percent | N | Percent | N | Percent |
| PSS * agecat2 | 157 | 100.0% | 0 | 0.0% | 157 | 100.0% |

| **Report** | | | |
| --- | --- | --- | --- |
| PSS | | | |
| agecat2 | Mean | N | Std. Deviation |
| 20-29years | 19.01 | 115 | 5.428 |
| 30-39years | 17.55 | 29 | 4.702 |
| 40 above | 14.31 | 13 | 5.154 |
| Total | 18.35 | 157 | 5.417 |

**Means**

| **Case Processing Summary** | | | | | | |
| --- | --- | --- | --- | --- | --- | --- |
|  | Cases | | | | | |
|  | Included | | Excluded | | Total | |
|  | N | Percent | N | Percent | N | Percent |
| PSS * desig3 | 157 | 100.0% | 0 | 0.0% | 157 | 100.0% |

| **Report** | | | |
| --- | --- | --- | --- |
| PSS | | | |
| desig3 | Mean | N | Std. Deviation |
| house officers | 18.93 | 60 | 5.099 |
| PG | 18.67 | 24 | 4.724 |
| lect and DP | 18.75 | 57 | 5.336 |
| AP and above | 14.25 | 16 | 6.506 |
| Total | 18.35 | 157 | 5.417 |
